# Supplementary figures and images for: Amino acid transporter SLC38A5 regulates developmental and pathological retinal angiogenesis
Source: eLife. 2022 Dec 1;11:e73105. doi: 10.7554/eLife.73105 (PMC9714971; doi:10.7554/eLife.73105)

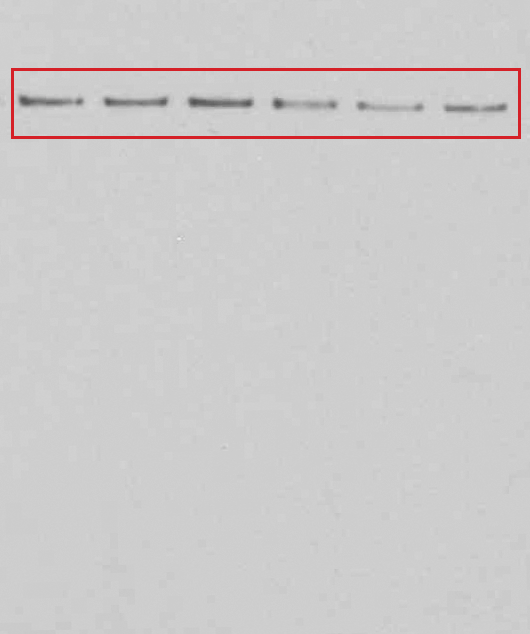

Supplement: Figure 1—source data 1. [file elife-73105-fig1-data1.zip › Figure 1-source data/Fig 1E/Fig1E WT and Lrp5 KO-SLC38A5-labeled.tif]

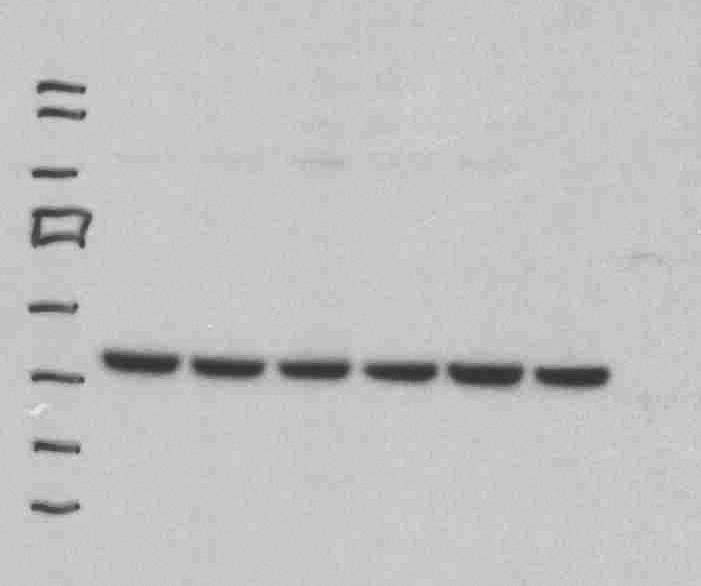

Supplement: Figure 1—source data 1. [file elife-73105-fig1-data1.zip › Figure 1-source data/Fig 1E/Fig1E WT and Lrp5 KO-GAPDH.tif]

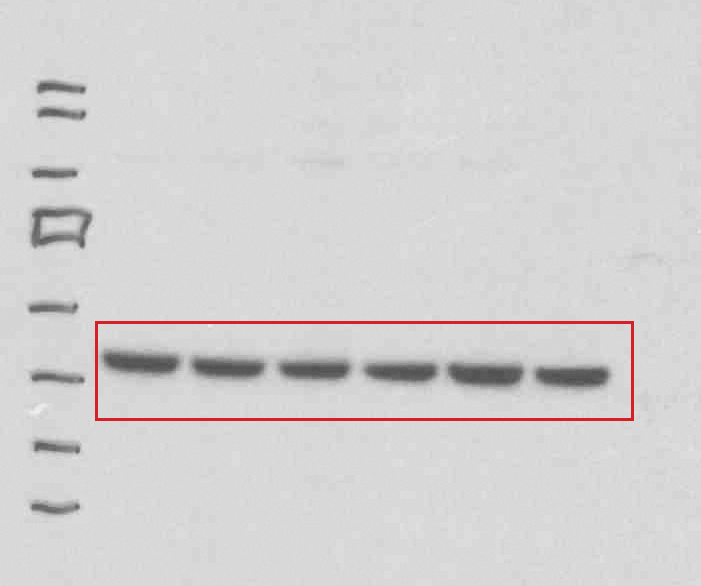

Supplement: Figure 1—source data 1. [file elife-73105-fig1-data1.zip › Figure 1-source data/Fig 1E/Fig1E WT and Lrp5 KO-GAPDH-labeled.tif]

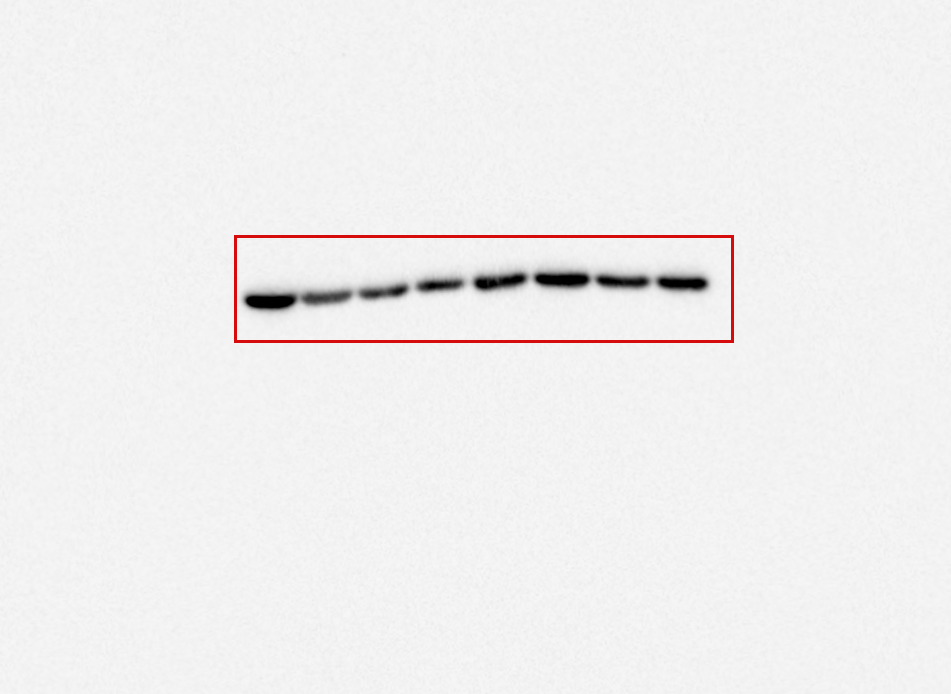

Supplement: Figure 1—source data 1. [file elife-73105-fig1-data1.zip › Figure 1-source data/Fig 1E/Fig1E Ndp WT and Ndp KO-GAPDH-labeled.tif]

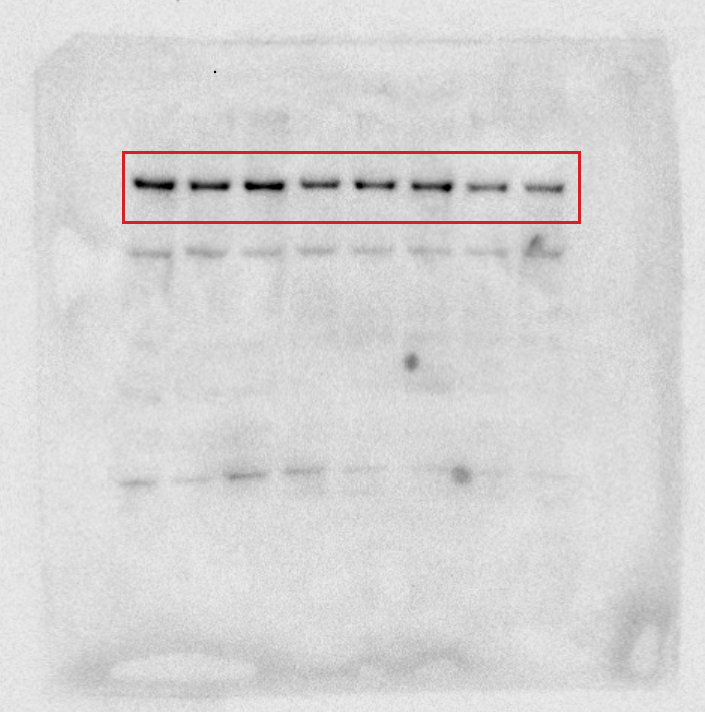

Supplement: Figure 1—source data 1. [file elife-73105-fig1-data1.zip › Figure 1-source data/Fig 1E/Fig1E Ndp WT and Ndp KO-SLC38A5-labeled.tif]

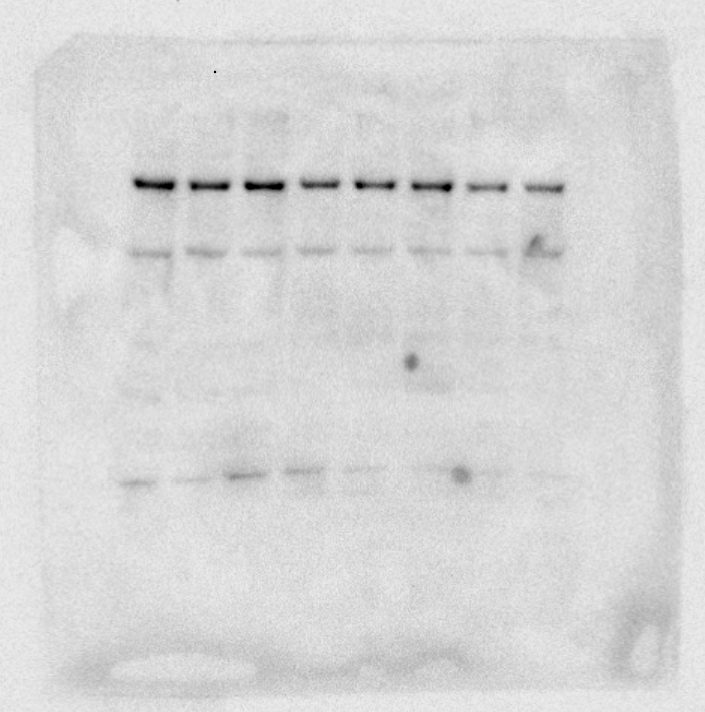

Supplement: Figure 1—source data 1. [file elife-73105-fig1-data1.zip › Figure 1-source data/Fig 1E/Fig1E Ndp WT and Ndp KO-SLC38A5.tif]

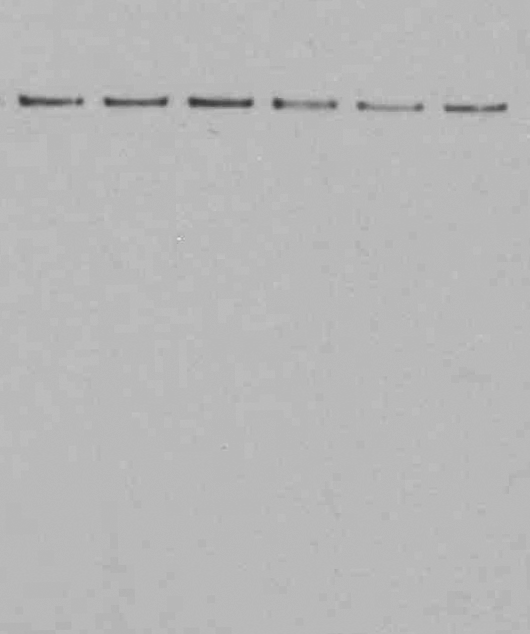

Supplement: Figure 1—source data 1. [file elife-73105-fig1-data1.zip › Figure 1-source data/Fig 1E/Fig1E WT and Lrp5 KO-SLC38A5.tif]

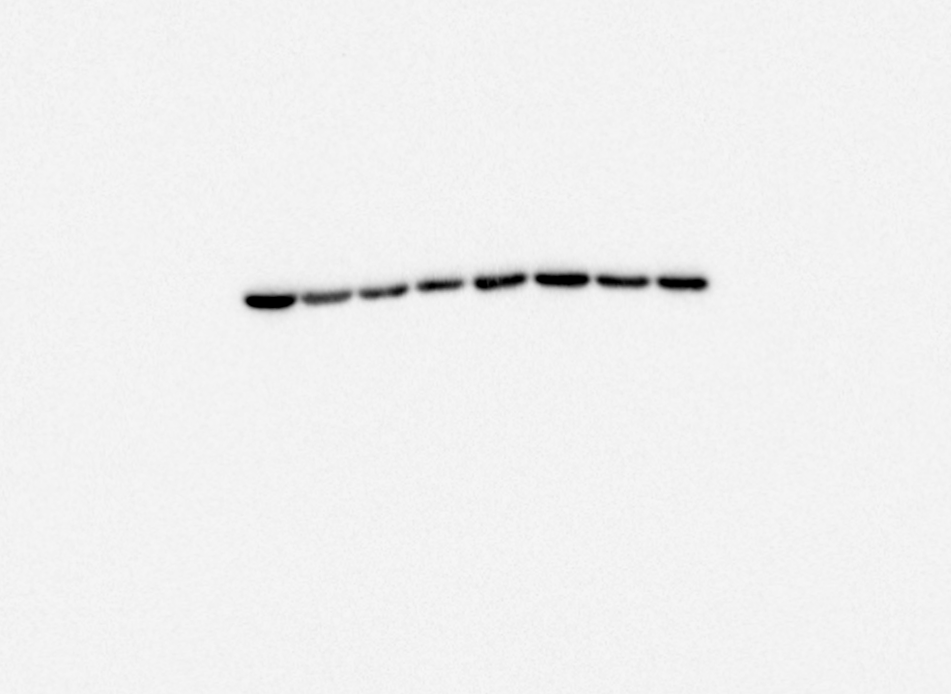

Supplement: Figure 1—source data 1. [file elife-73105-fig1-data1.zip › Figure 1-source data/Fig 1E/Fig1E Ndp WT and Ndp KO-GAPDH.tif]

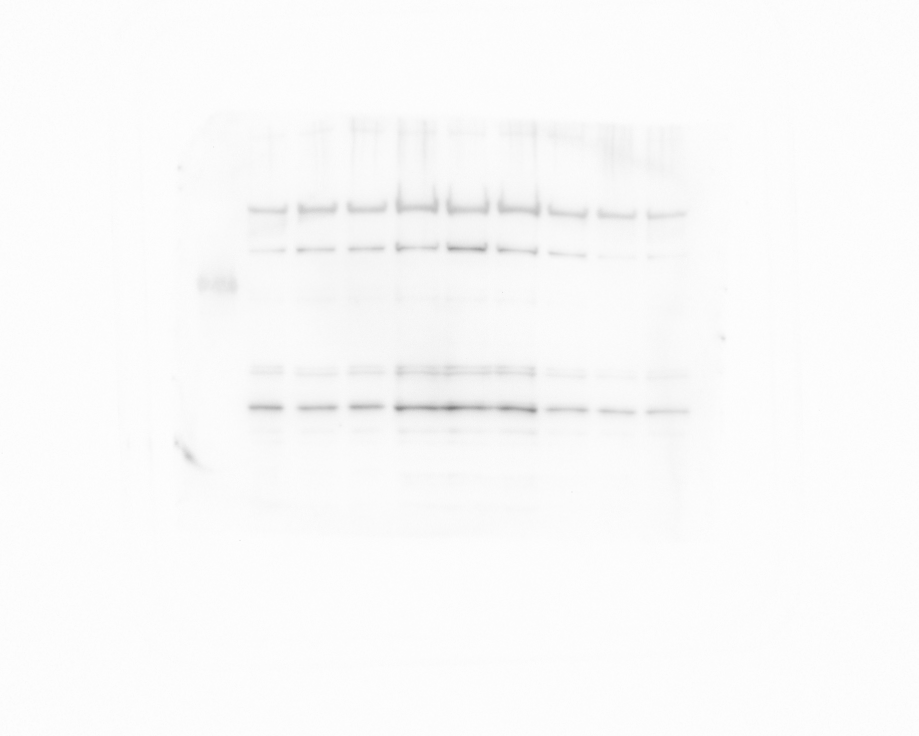

Supplement: Figure 2—source data 1. [file elife-73105-fig2-data1.zip › Figure 2-source data/Fig 2B/Fig2B WB-SLC38A5.tif]

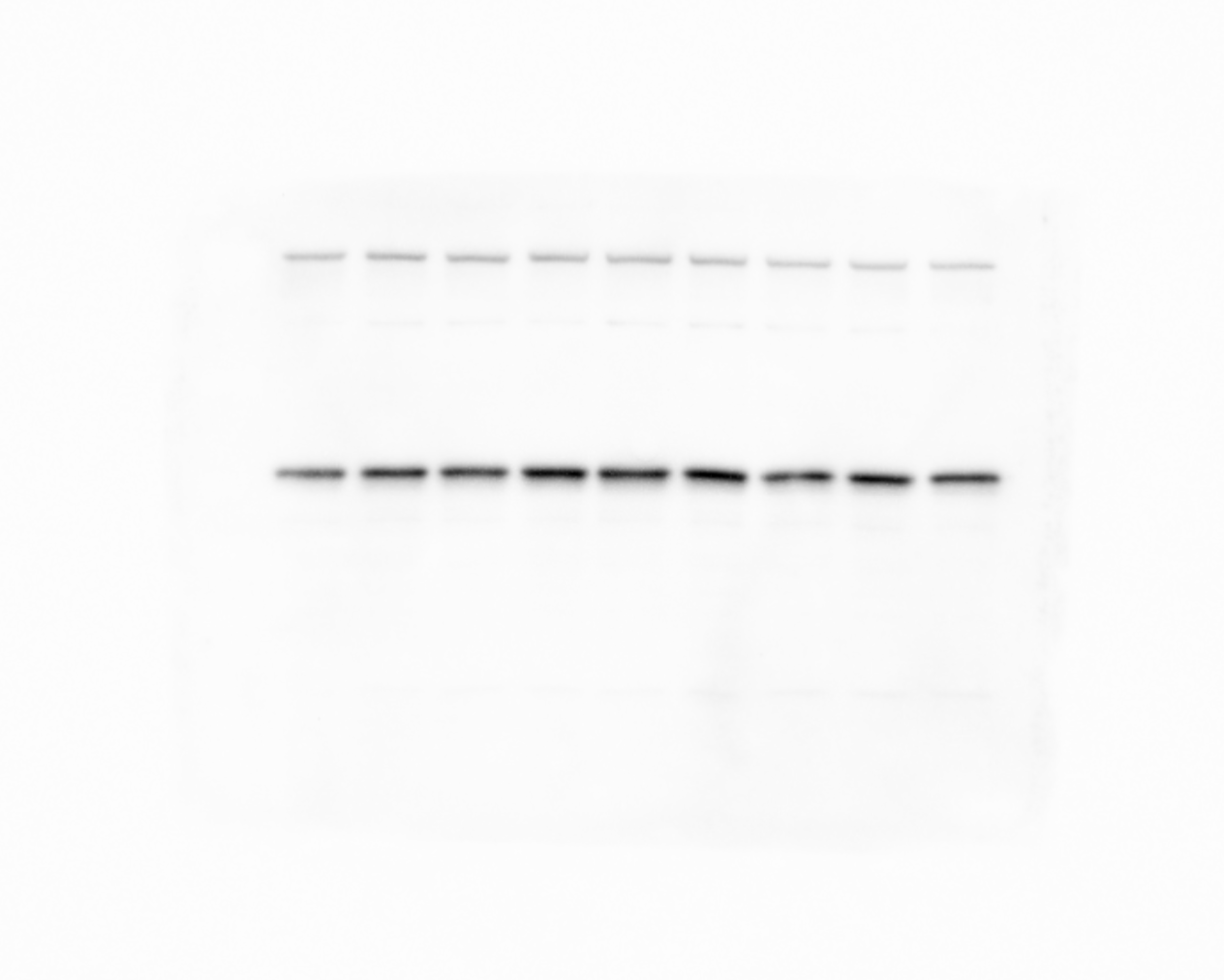

Supplement: Figure 2—source data 1. [file elife-73105-fig2-data1.zip › Figure 2-source data/Fig 2B/Fig2B WB-n-p-b-catenin.tif]

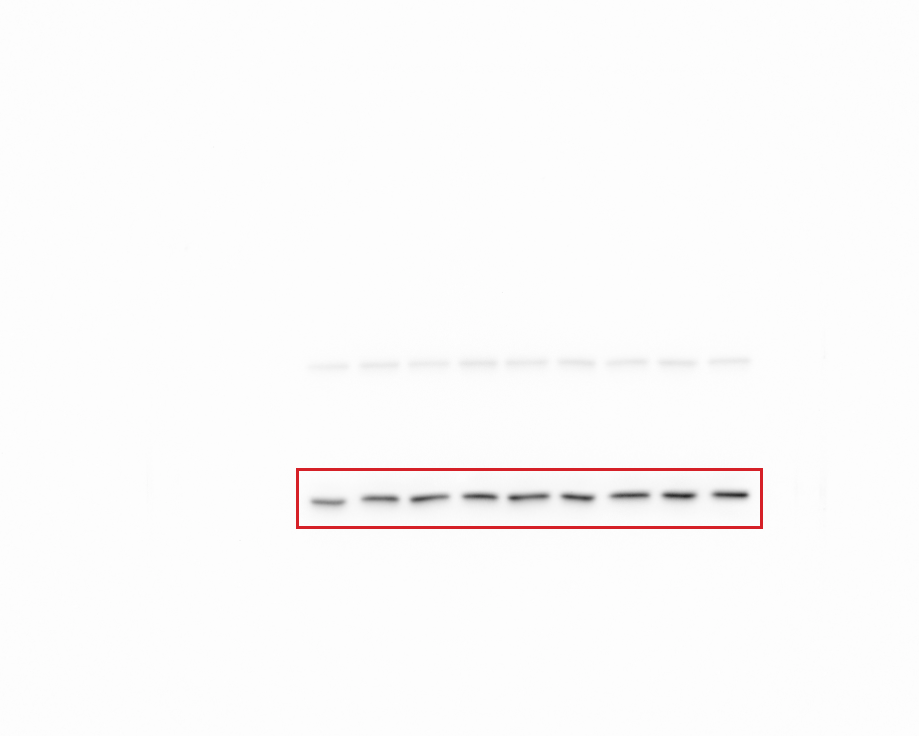

Supplement: Figure 2—source data 1. [file elife-73105-fig2-data1.zip › Figure 2-source data/Fig 2B/Fig2B WB-GAPDH-labeled.tif]

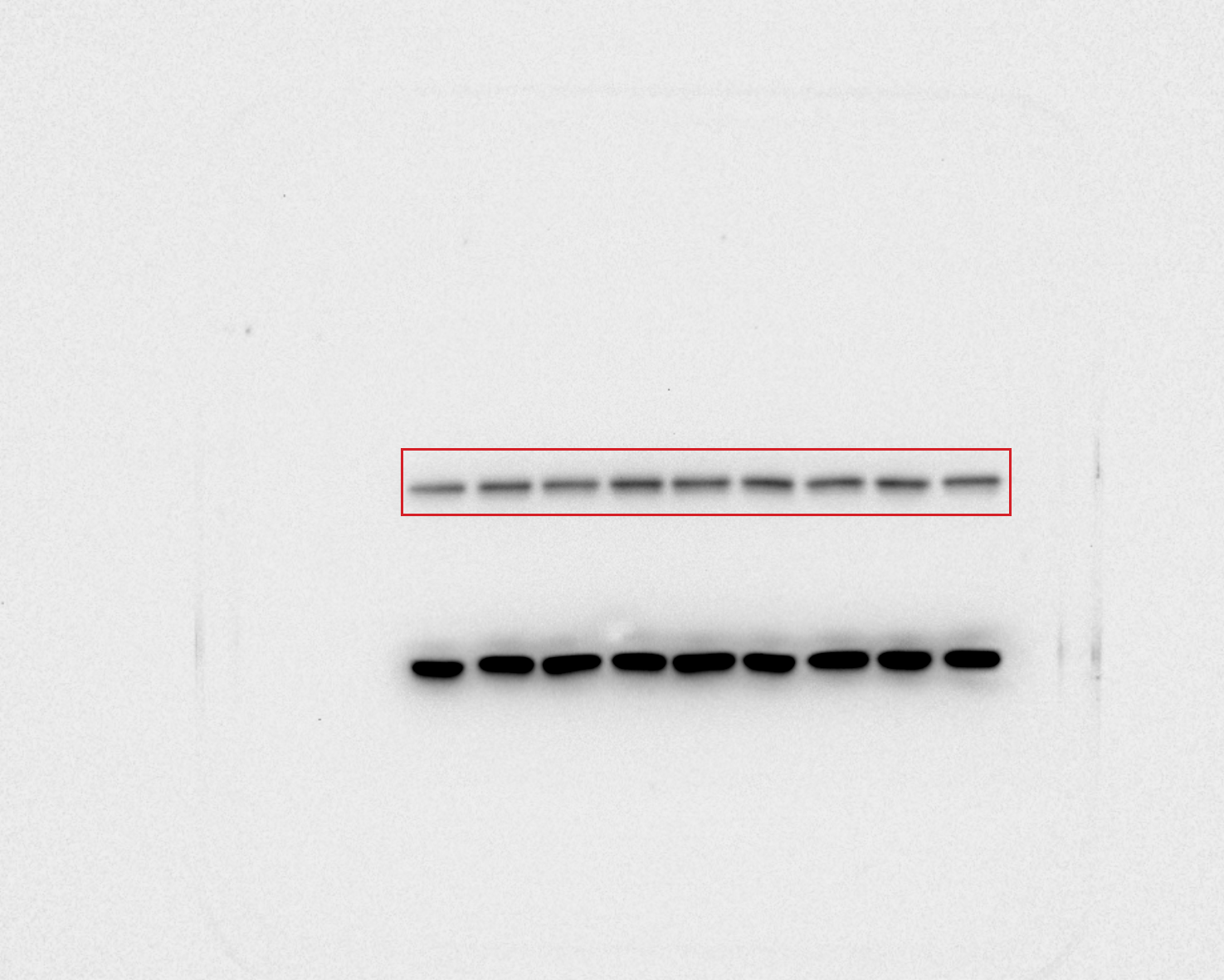

Supplement: Figure 2—source data 1. [file elife-73105-fig2-data1.zip › Figure 2-source data/Fig 2B/Fig2B WB-b-catenin-labeled.tif]

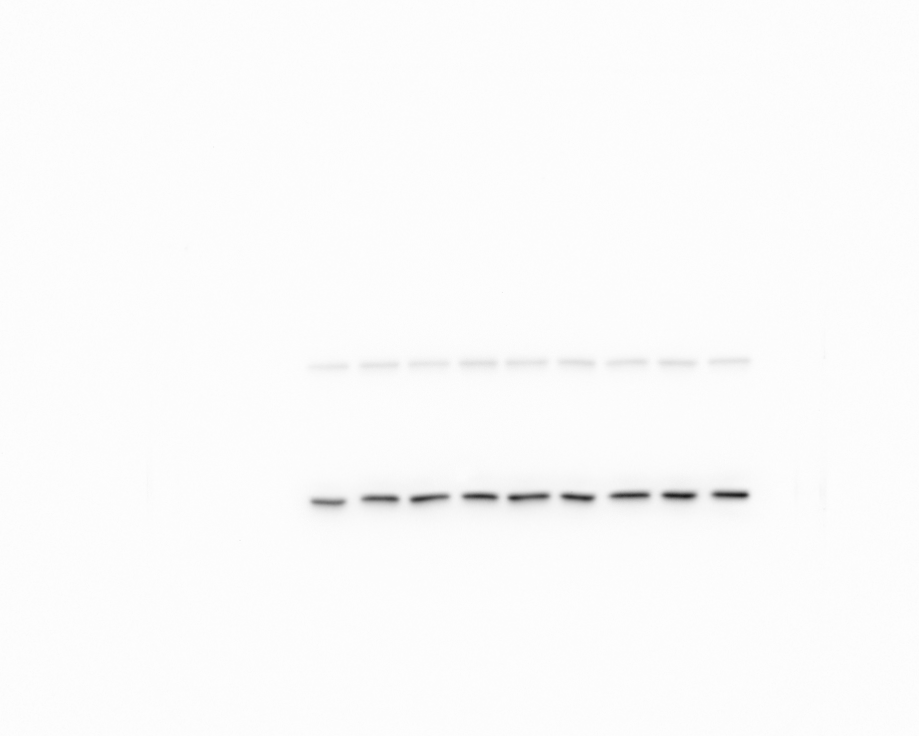

Supplement: Figure 2—source data 1. [file elife-73105-fig2-data1.zip › Figure 2-source data/Fig 2B/Fig2B WB-GAPDH.tif]

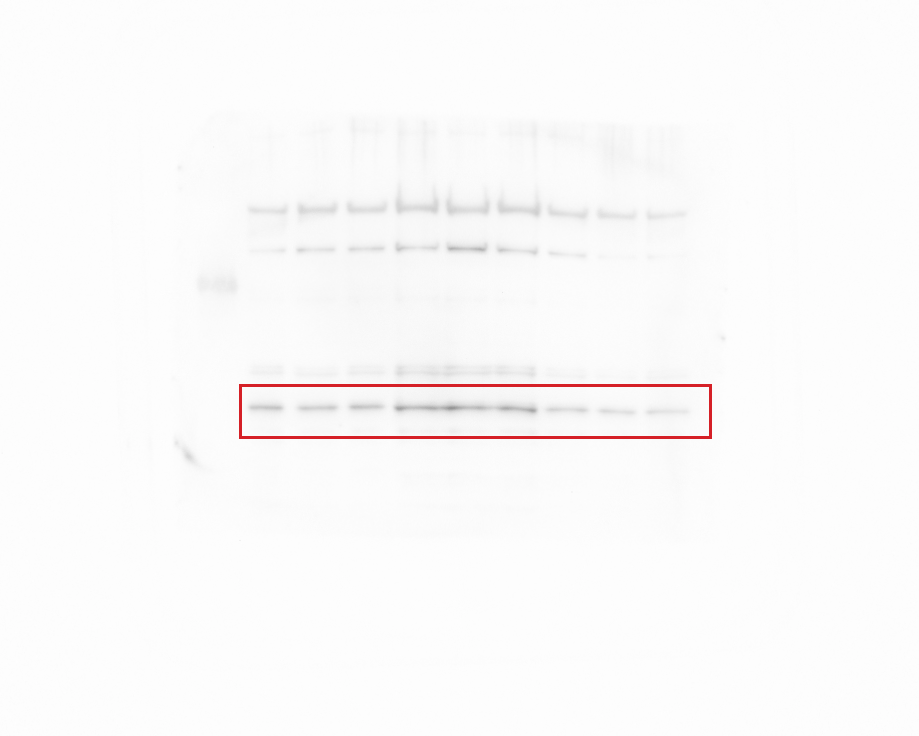

Supplement: Figure 2—source data 1. [file elife-73105-fig2-data1.zip › Figure 2-source data/Fig 2B/Fig2B WB-SLC38A5-labeled.tif]

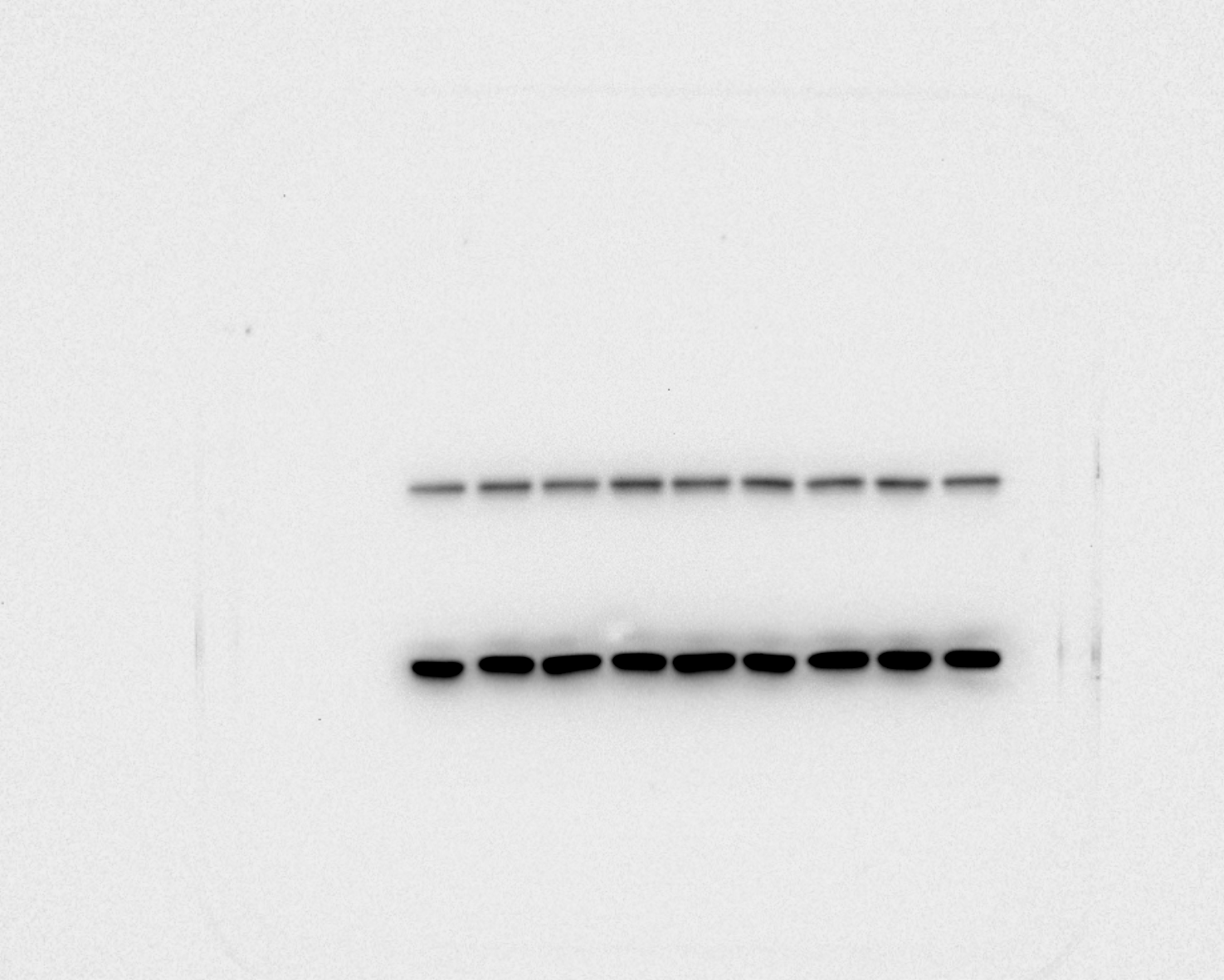

Supplement: Figure 2—source data 1. [file elife-73105-fig2-data1.zip › Figure 2-source data/Fig 2B/Fig2B WB-b-catenin.tif]

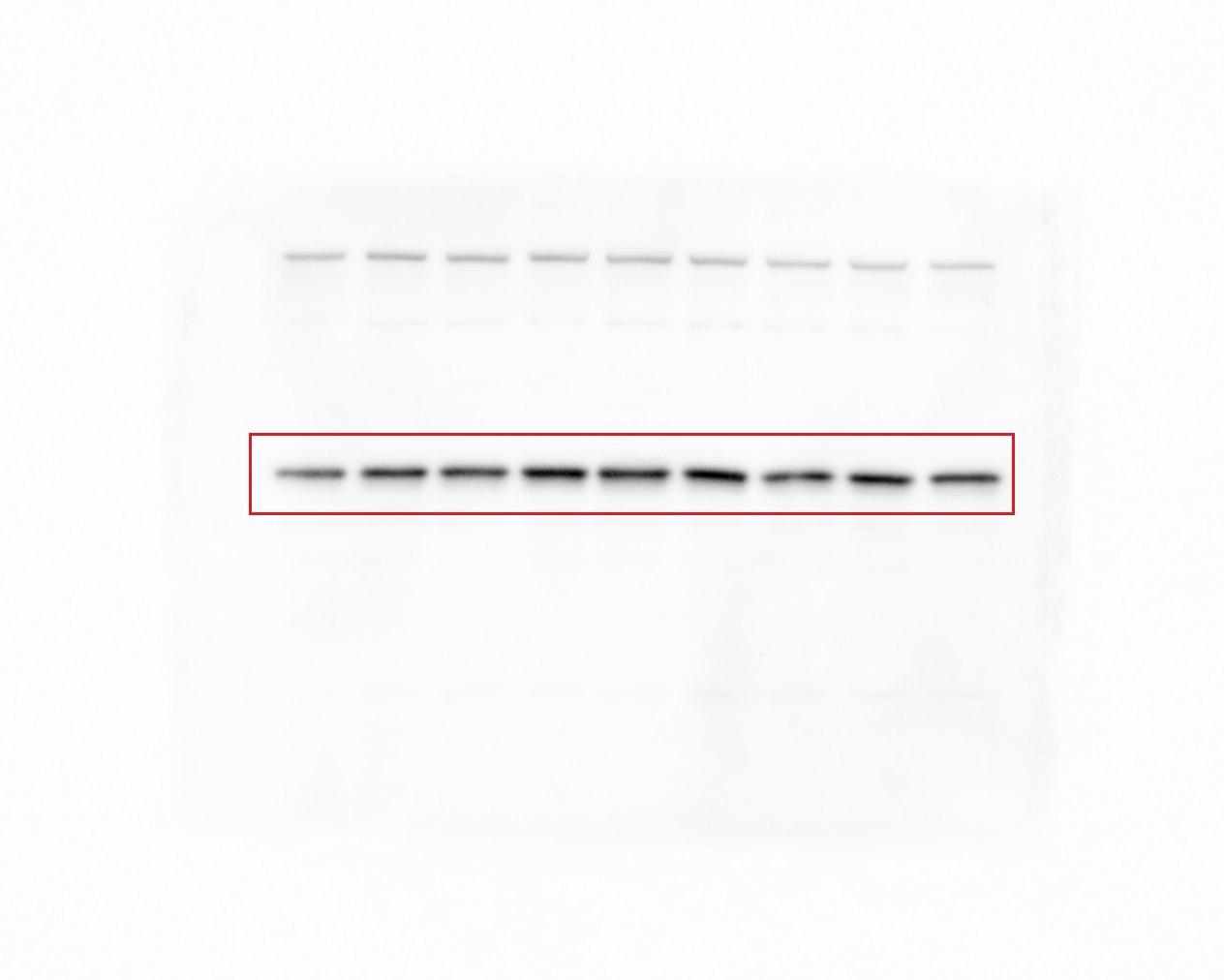

Supplement: Figure 2—source data 1. [file elife-73105-fig2-data1.zip › Figure 2-source data/Fig 2B/Fig2B WB-n-p-b-catenin-labeled.tif]

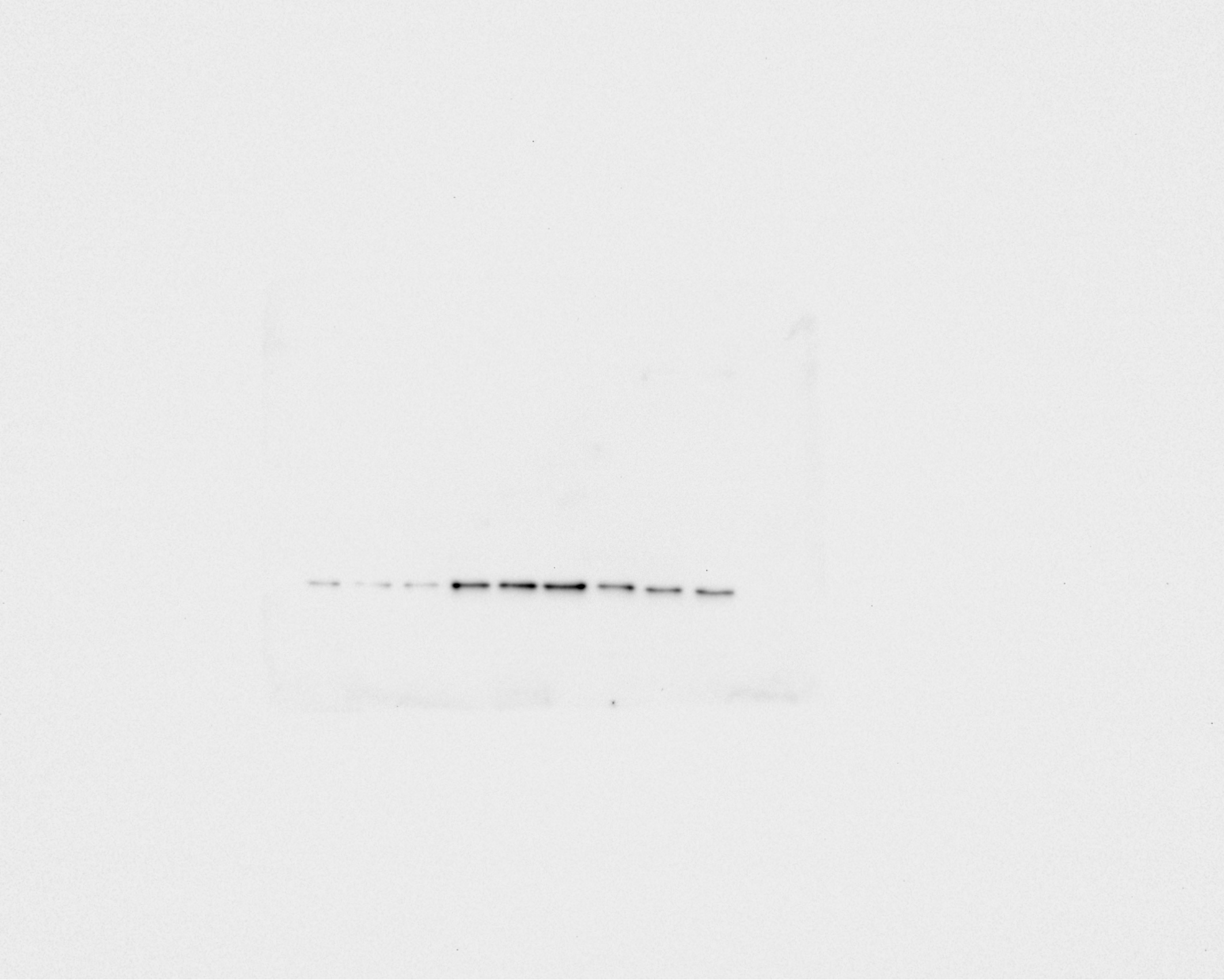

Supplement: Figure 2—source data 1. [file elife-73105-fig2-data1.zip › Figure 2-source data/Fig 2C/Fig2C_WB_SLC38A5.tif]

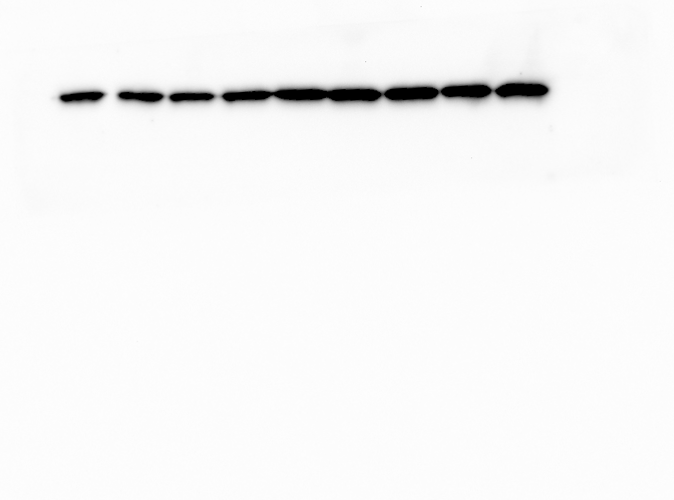

Supplement: Figure 2—source data 1. [file elife-73105-fig2-data1.zip › Figure 2-source data/Fig 2C/Fig2C_WB_b-catenin.tif]

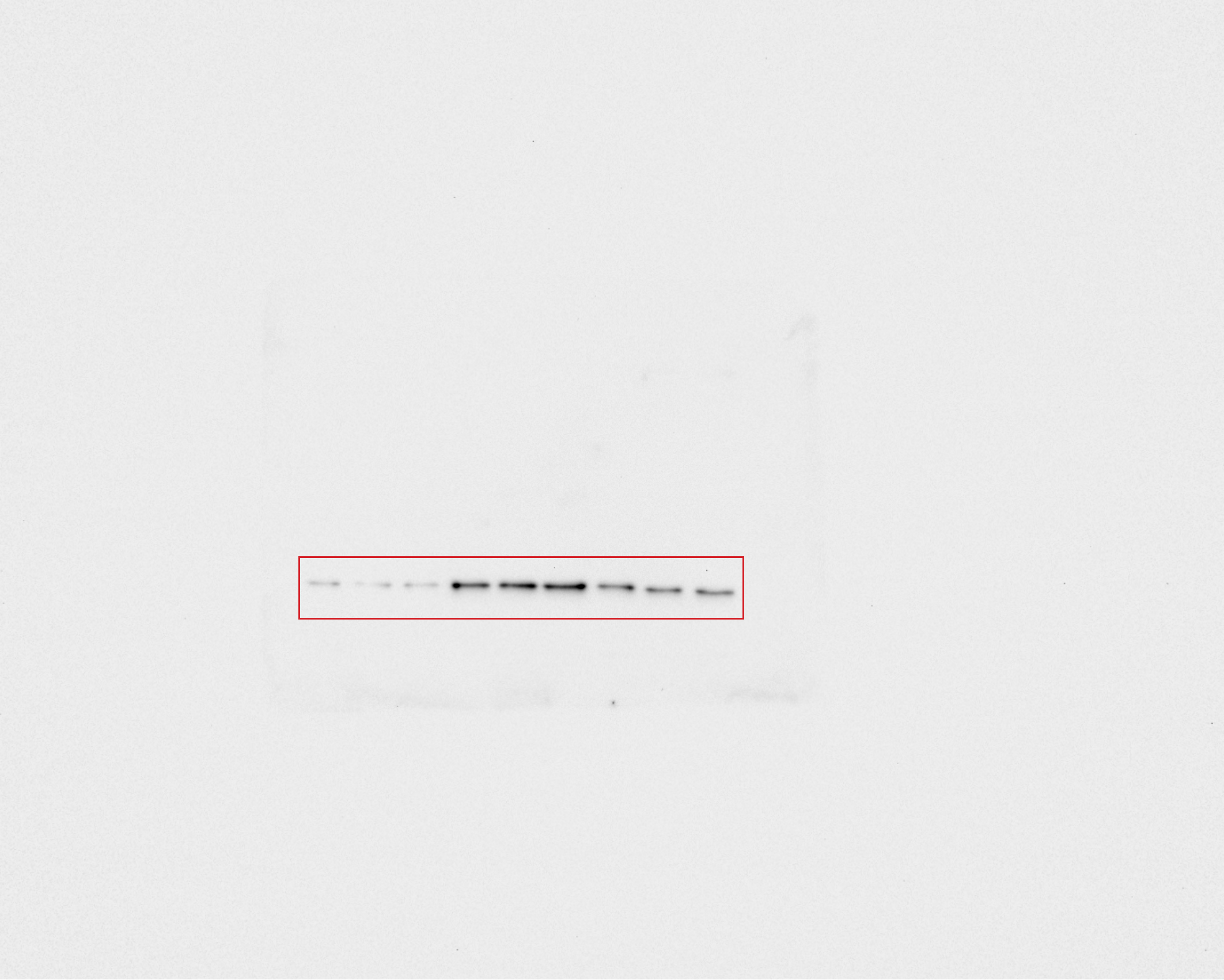

Supplement: Figure 2—source data 1. [file elife-73105-fig2-data1.zip › Figure 2-source data/Fig 2C/Fig2C_WB_SLC38A5-labeled.tif]

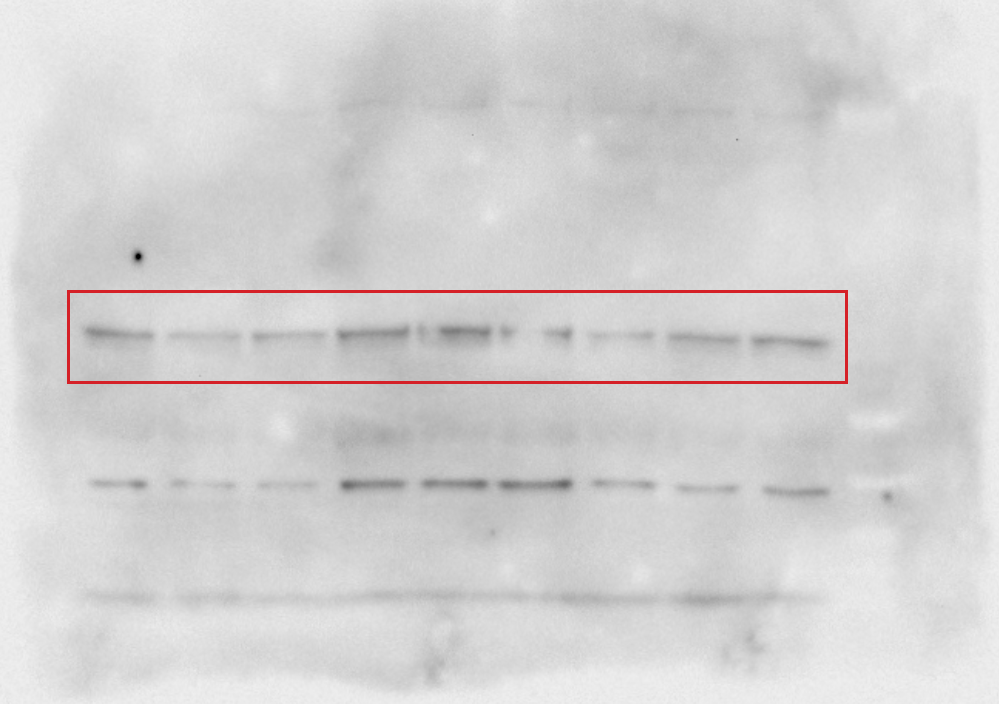

Supplement: Figure 2—source data 1. [file elife-73105-fig2-data1.zip › Figure 2-source data/Fig 2C/Fig2C_WB_n-p-b-catenin-labeled.tif]

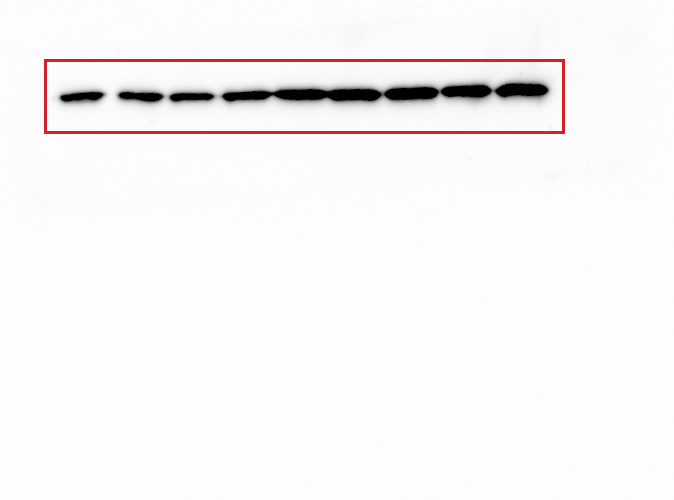

Supplement: Figure 2—source data 1. [file elife-73105-fig2-data1.zip › Figure 2-source data/Fig 2C/Fig2C_WB_b-catenin-labeled.tif]

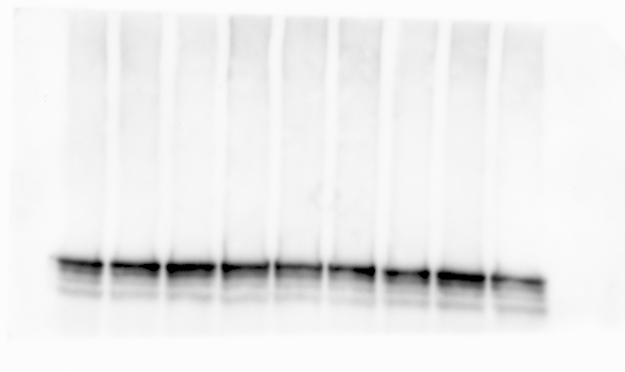

Supplement: Figure 2—source data 1. [file elife-73105-fig2-data1.zip › Figure 2-source data/Fig 2C/Fig2C_WB_GAPDH.tif]

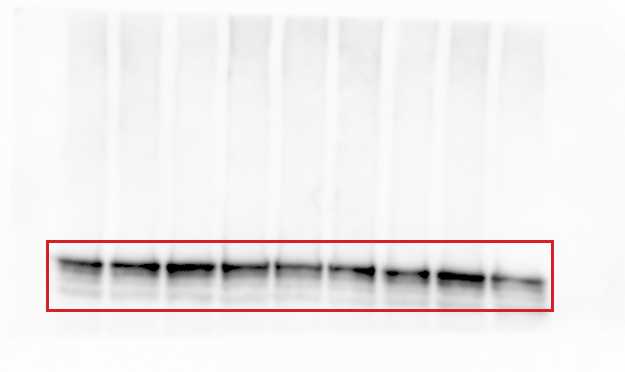

Supplement: Figure 2—source data 1. [file elife-73105-fig2-data1.zip › Figure 2-source data/Fig 2C/Fig2C_WB_GAPDH-labeled.tif]

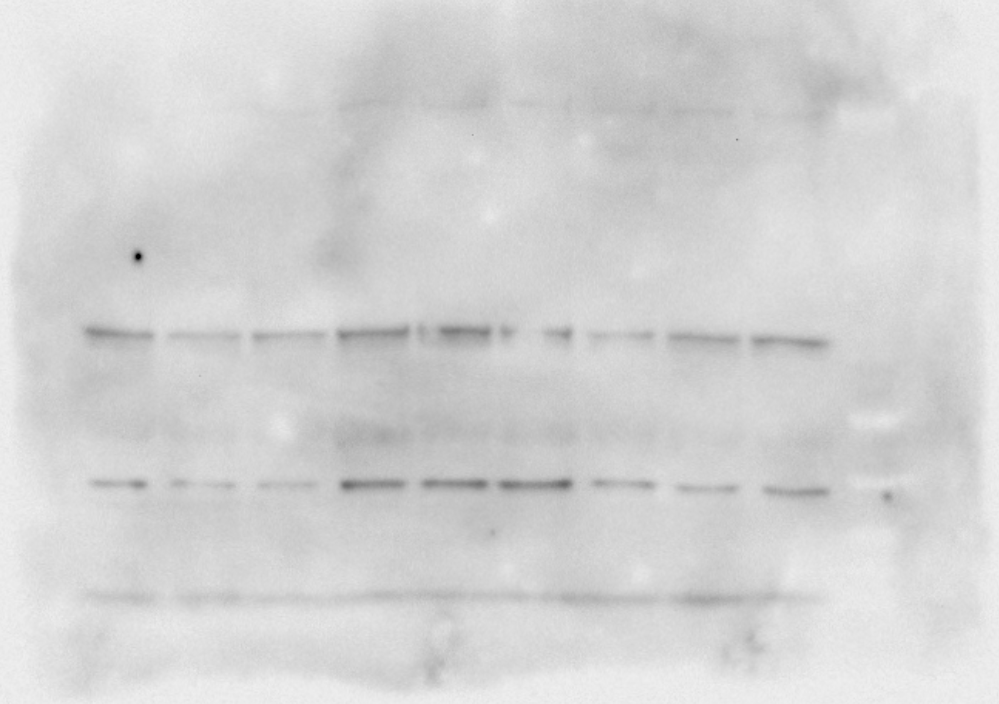

Supplement: Figure 2—source data 1. [file elife-73105-fig2-data1.zip › Figure 2-source data/Fig 2C/Fig2C_WB_n-p-b-catenin.tif]

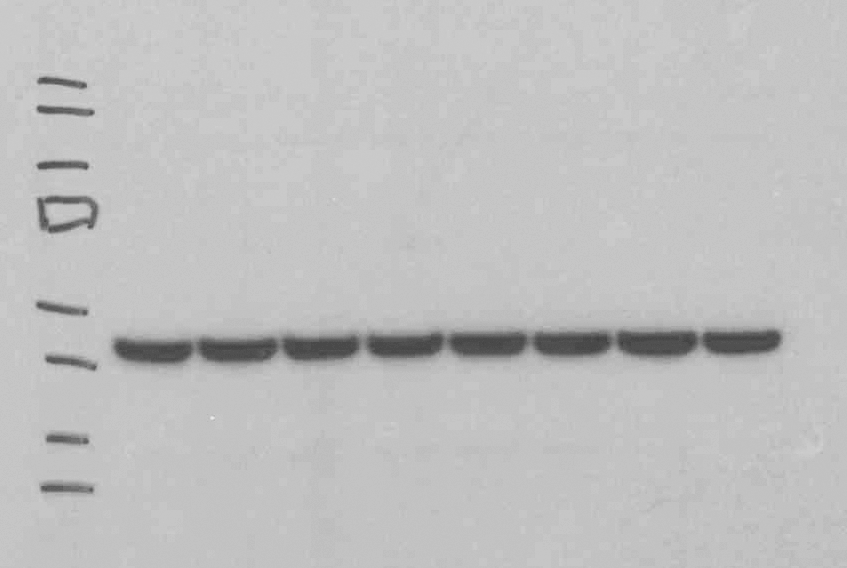

Supplement: Figure 3—source data 1. [file elife-73105-fig3-data1.zip › Figure 3-source data/Fig 3B/Fig3B_WB_GAPDH.tif]

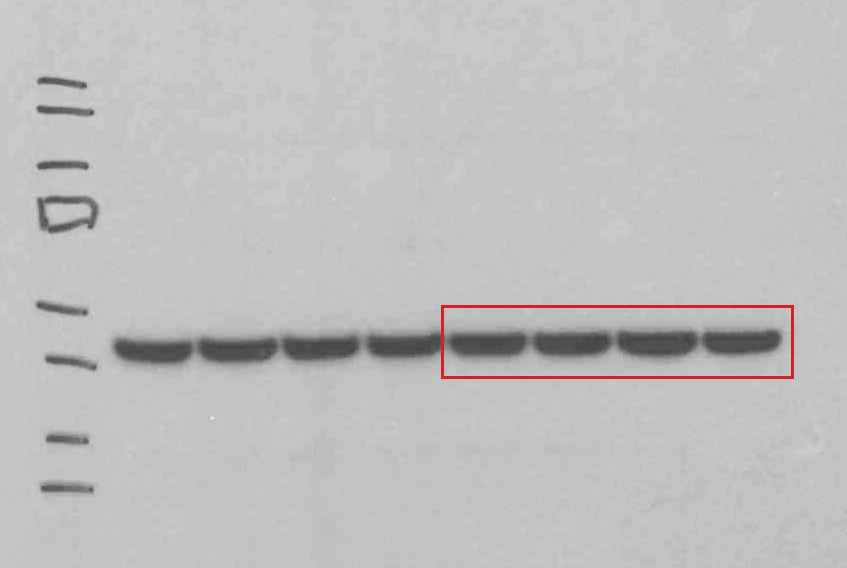

Supplement: Figure 3—source data 1. [file elife-73105-fig3-data1.zip › Figure 3-source data/Fig 3B/Fig3B_WB_GAPDH-labeled.tif]

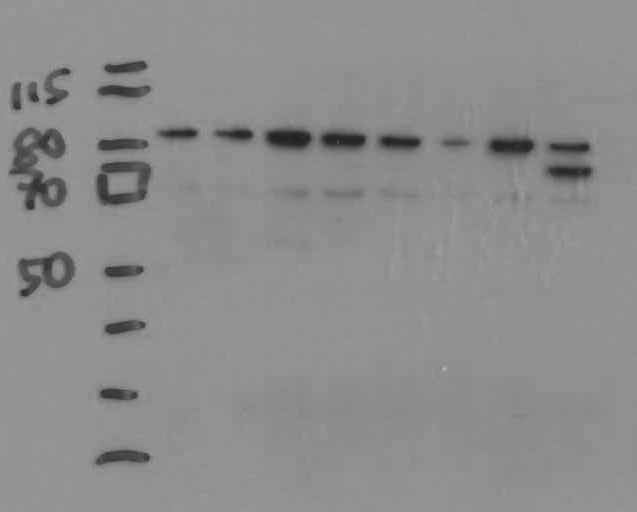

Supplement: Figure 3—source data 1. [file elife-73105-fig3-data1.zip › Figure 3-source data/Fig 3B/Fig3B_WB_SLC38A5.tif]

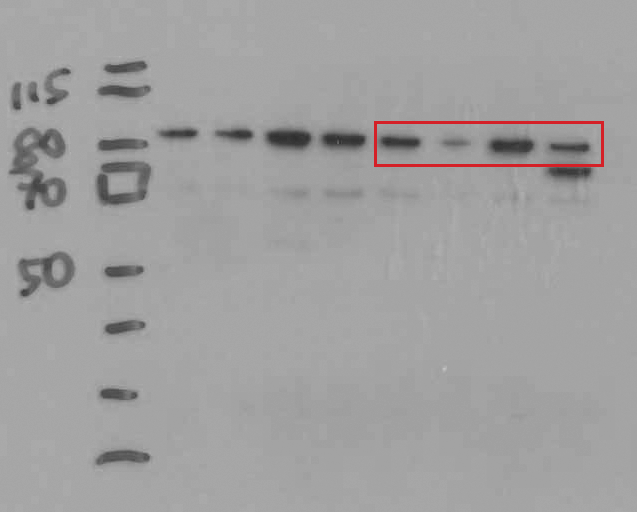

Supplement: Figure 3—source data 1. [file elife-73105-fig3-data1.zip › Figure 3-source data/Fig 3B/Fig3B_WB_SLC38A5-labeled.tif]

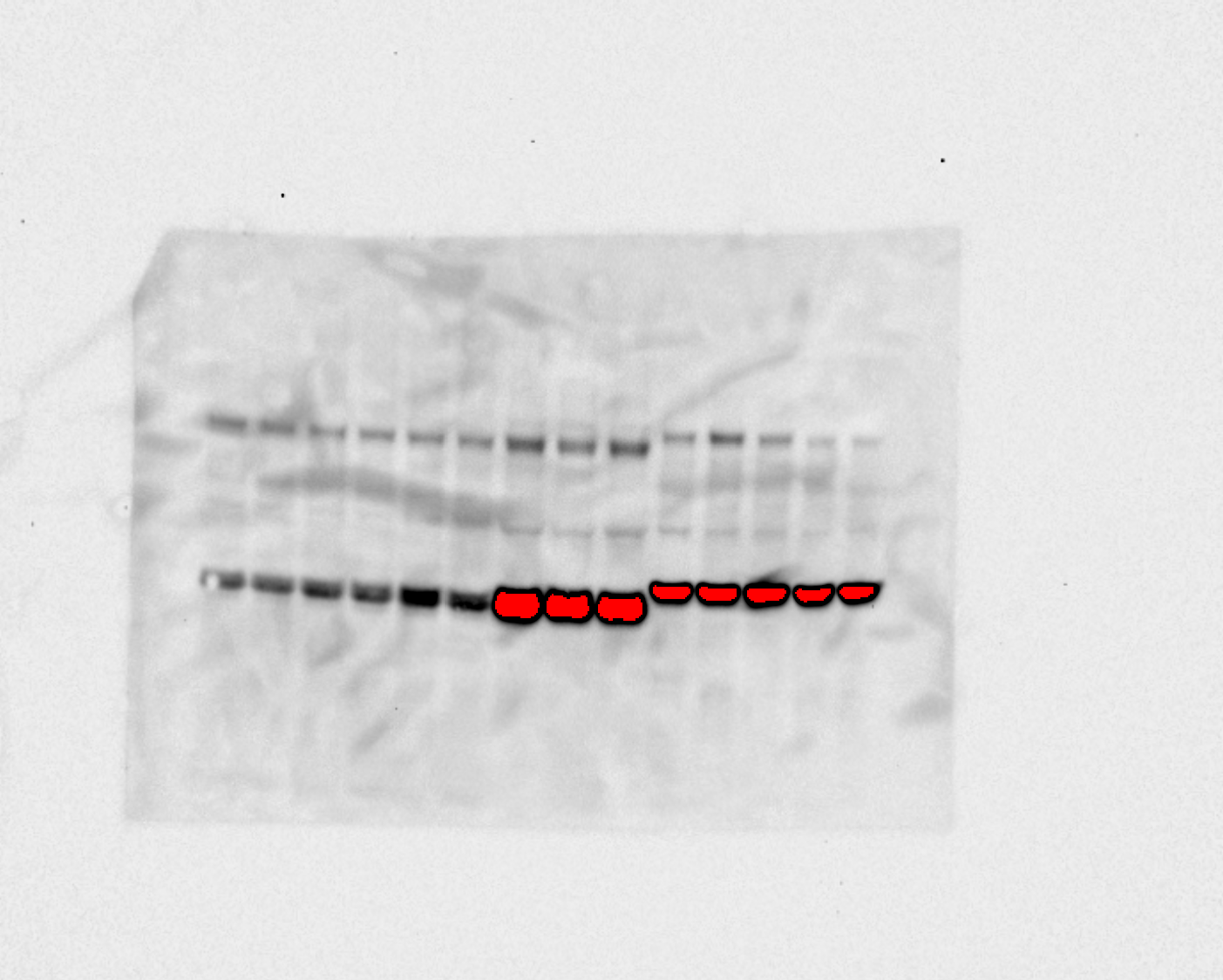

Supplement: Figure 4—source data 1. [file elife-73105-fig4-data1.zip › Figure 4-source data/Fig 4C/Fig4C_WB_b-actin.tif]

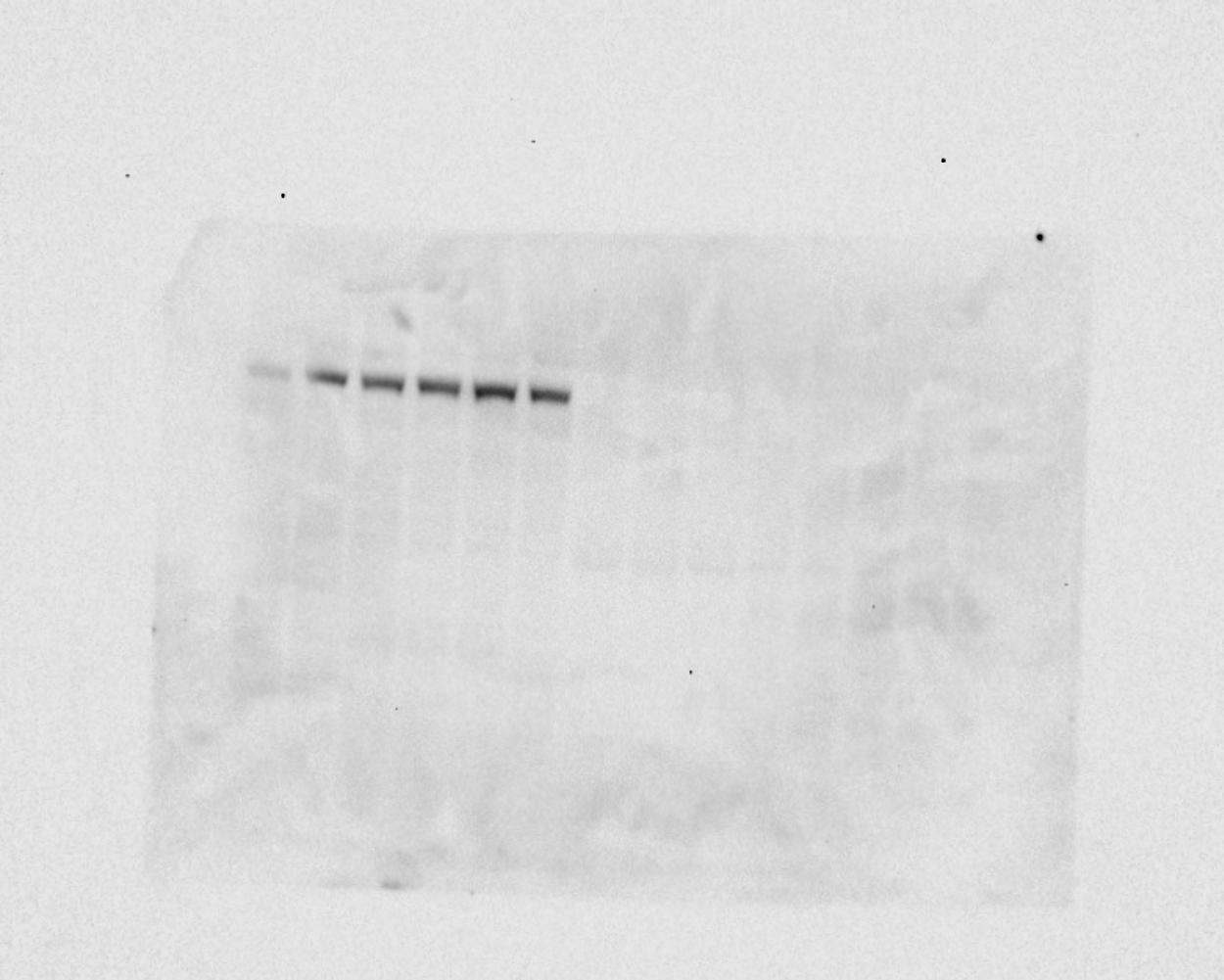

Supplement: Figure 4—source data 1. [file elife-73105-fig4-data1.zip › Figure 4-source data/Fig 4C/Fig4C_WB_Slc38a5.tif]

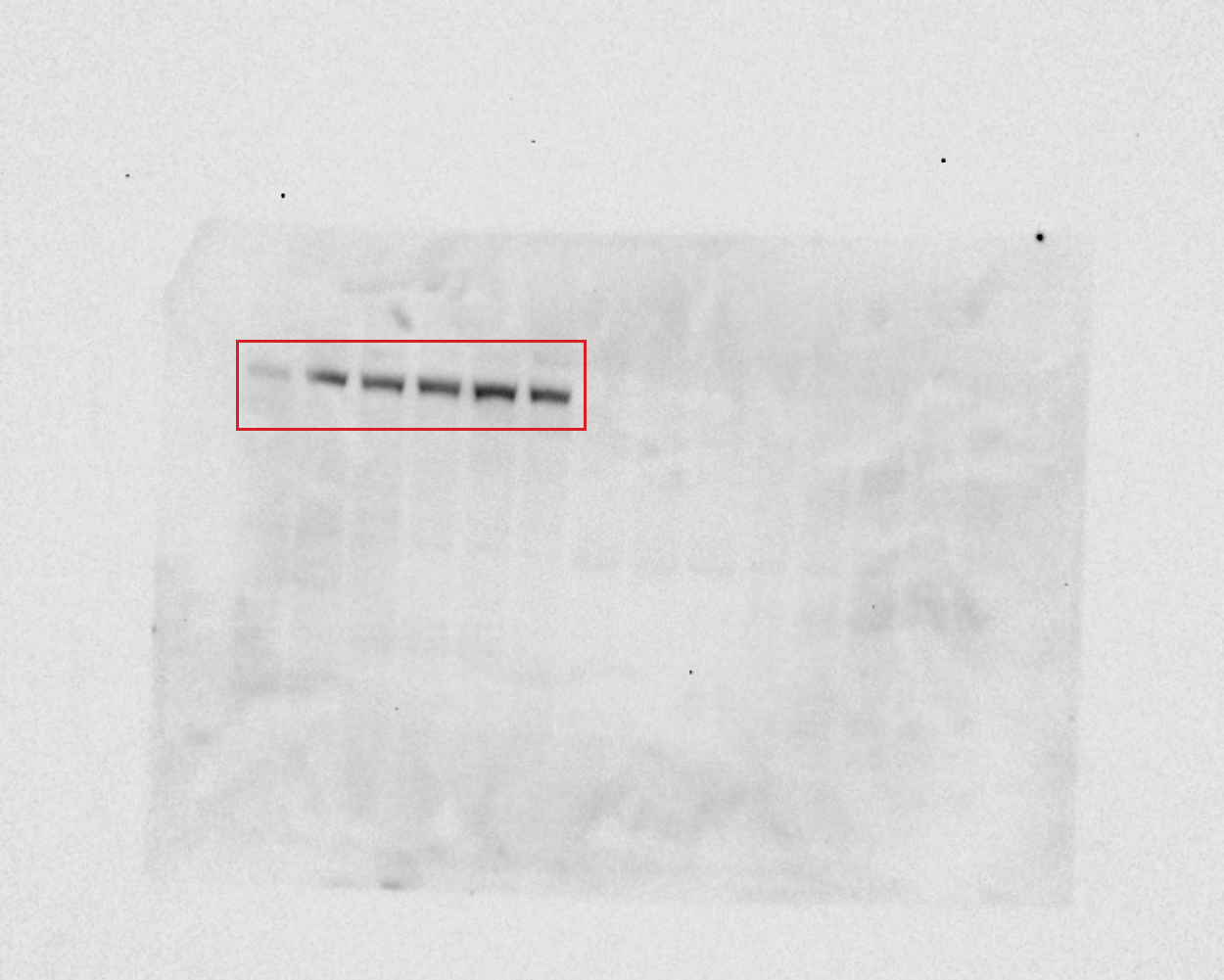

Supplement: Figure 4—source data 1. [file elife-73105-fig4-data1.zip › Figure 4-source data/Fig 4C/Fig4C_WB_Slc38a5-labeled.tif]

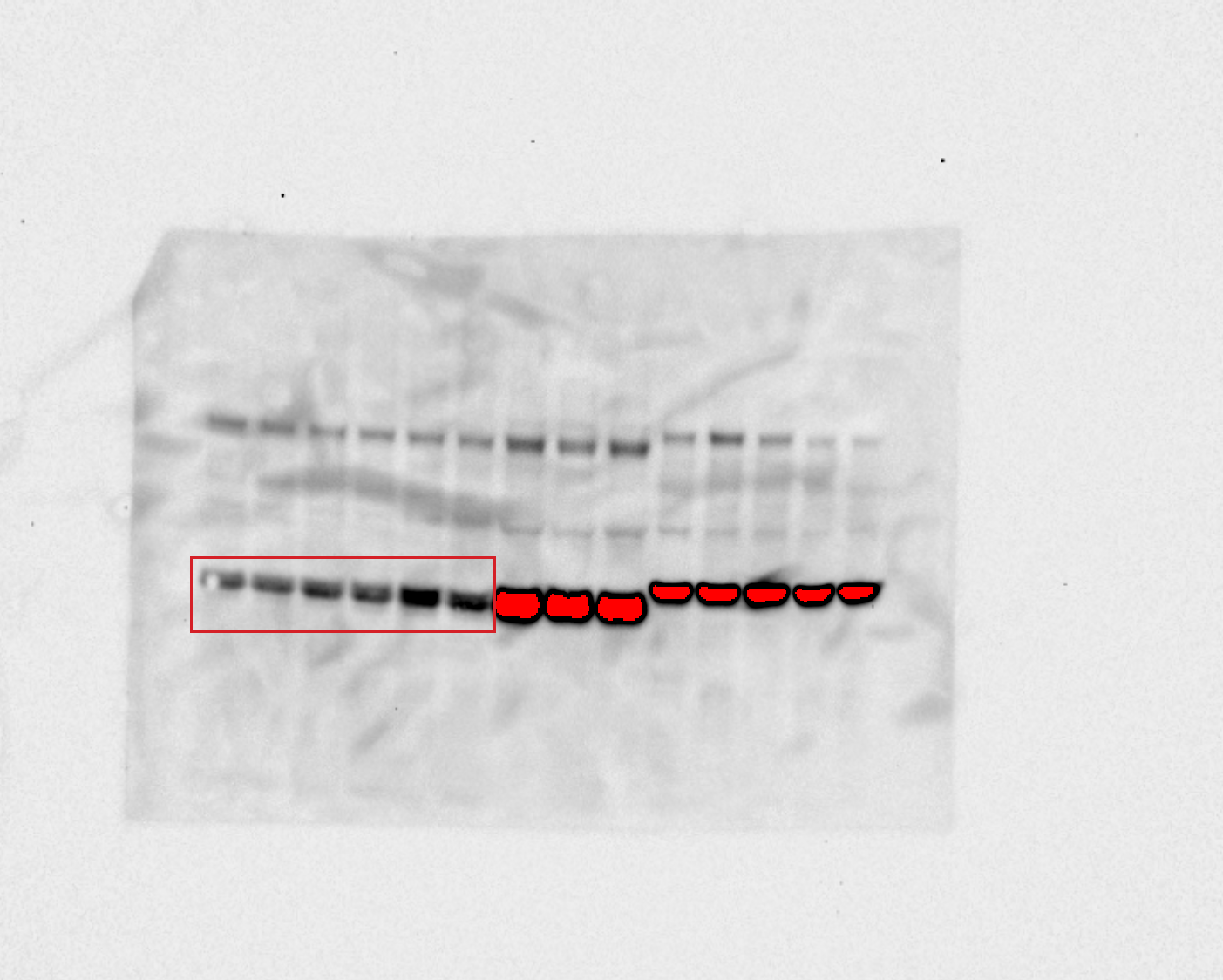

Supplement: Figure 4—source data 1. [file elife-73105-fig4-data1.zip › Figure 4-source data/Fig 4C/Fig4C_WB_b-actin-labeled.tif]

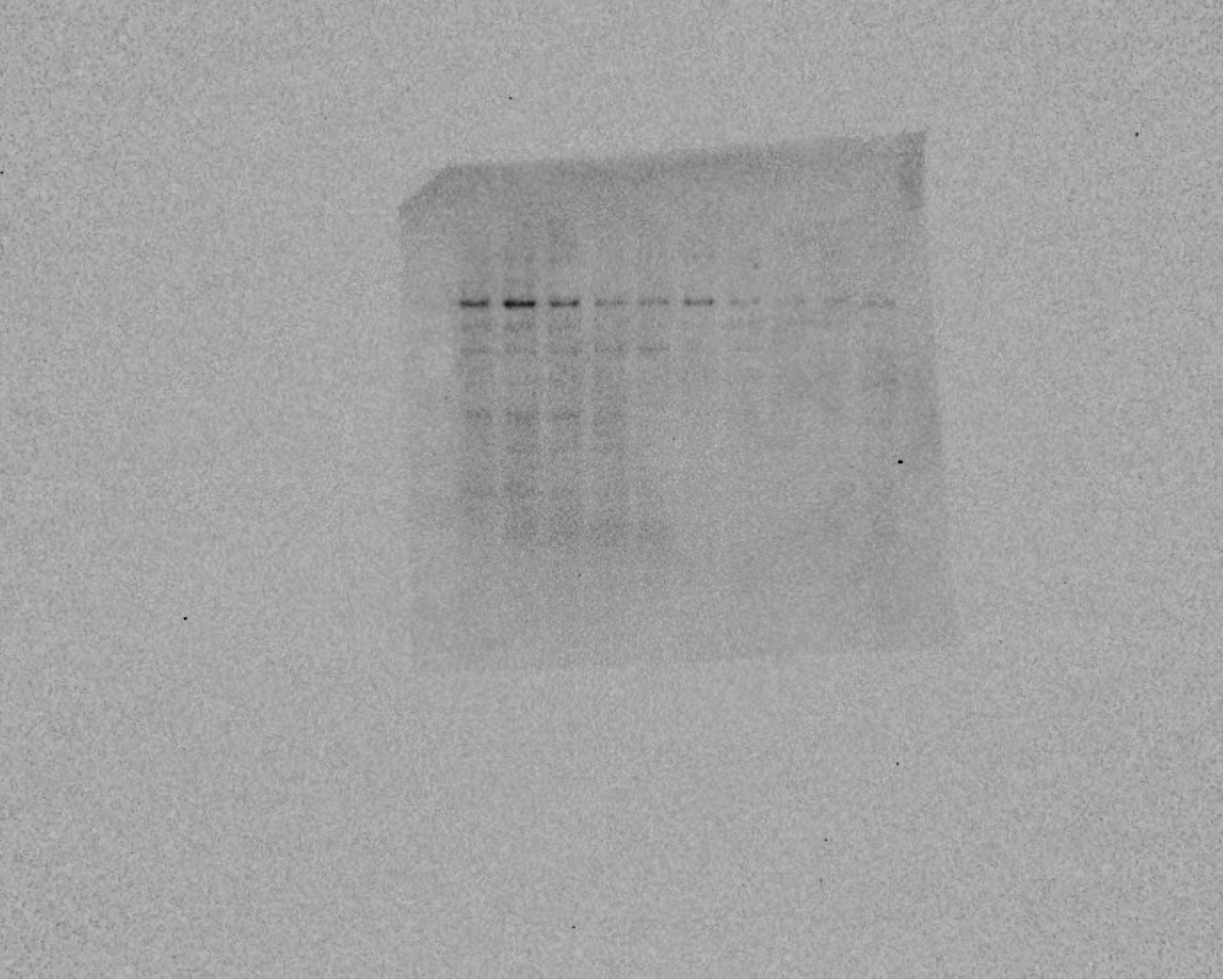

Supplement: Figure 5—source data 1. [file elife-73105-fig5-data1.zip › Figure 5-source data/Fig 5B/Fig5B_WB_Slc38a5.tif]

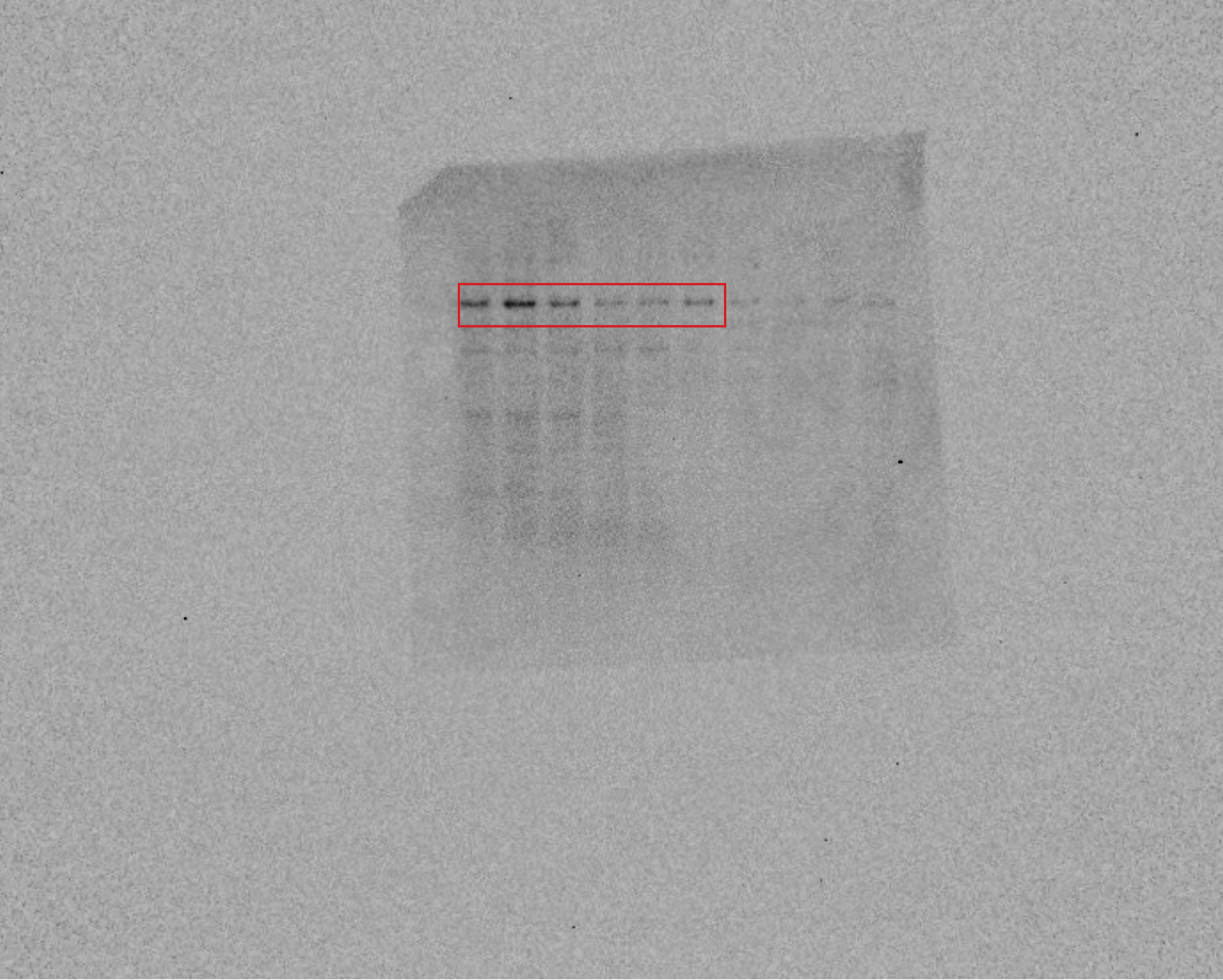

Supplement: Figure 5—source data 1. [file elife-73105-fig5-data1.zip › Figure 5-source data/Fig 5B/Fig5B_WB_Slc38a5-labeled.tif]

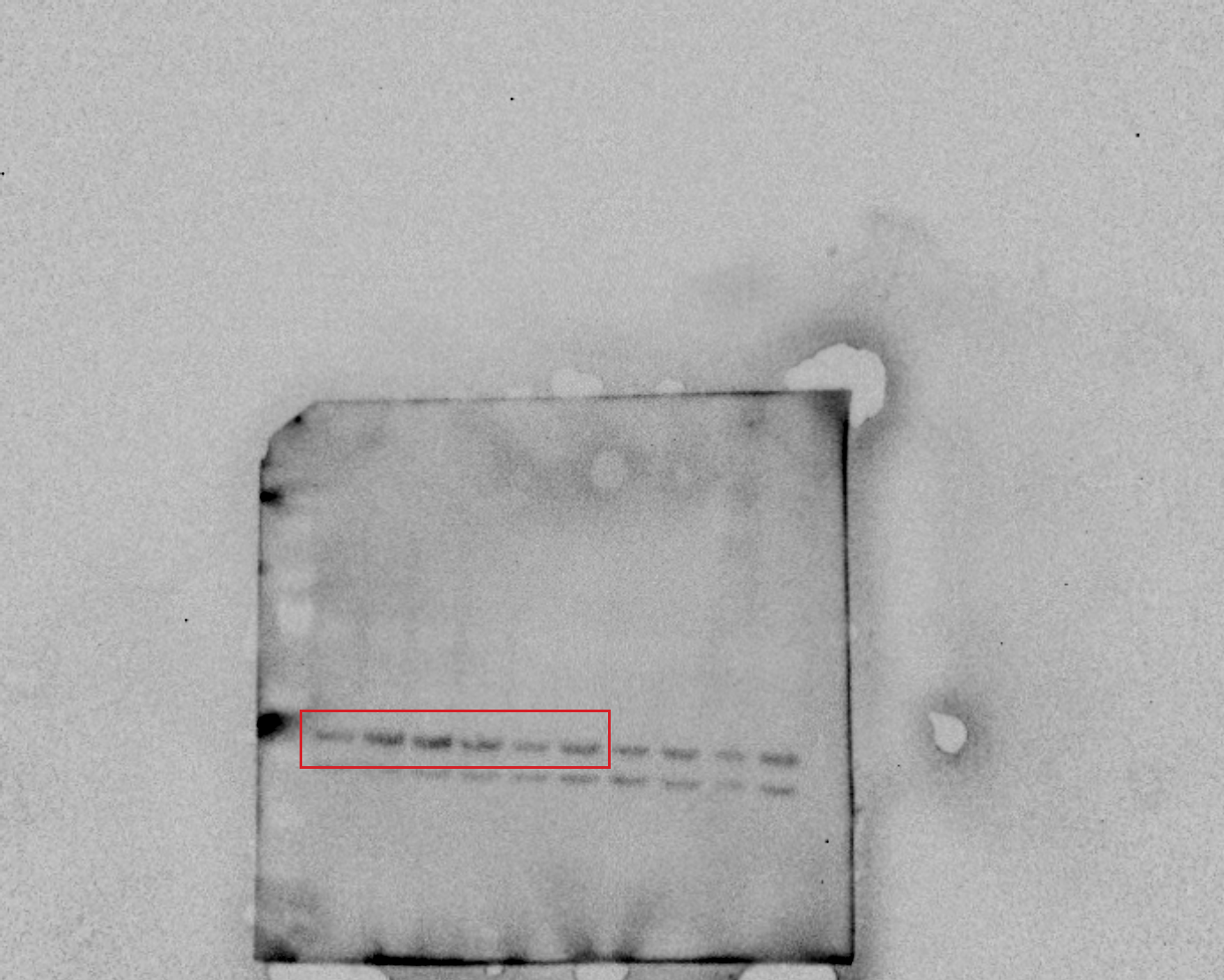

Supplement: Figure 5—source data 1. [file elife-73105-fig5-data1.zip › Figure 5-source data/Fig 5B/Fig5B_WB_GAPDH-labeled.tif]

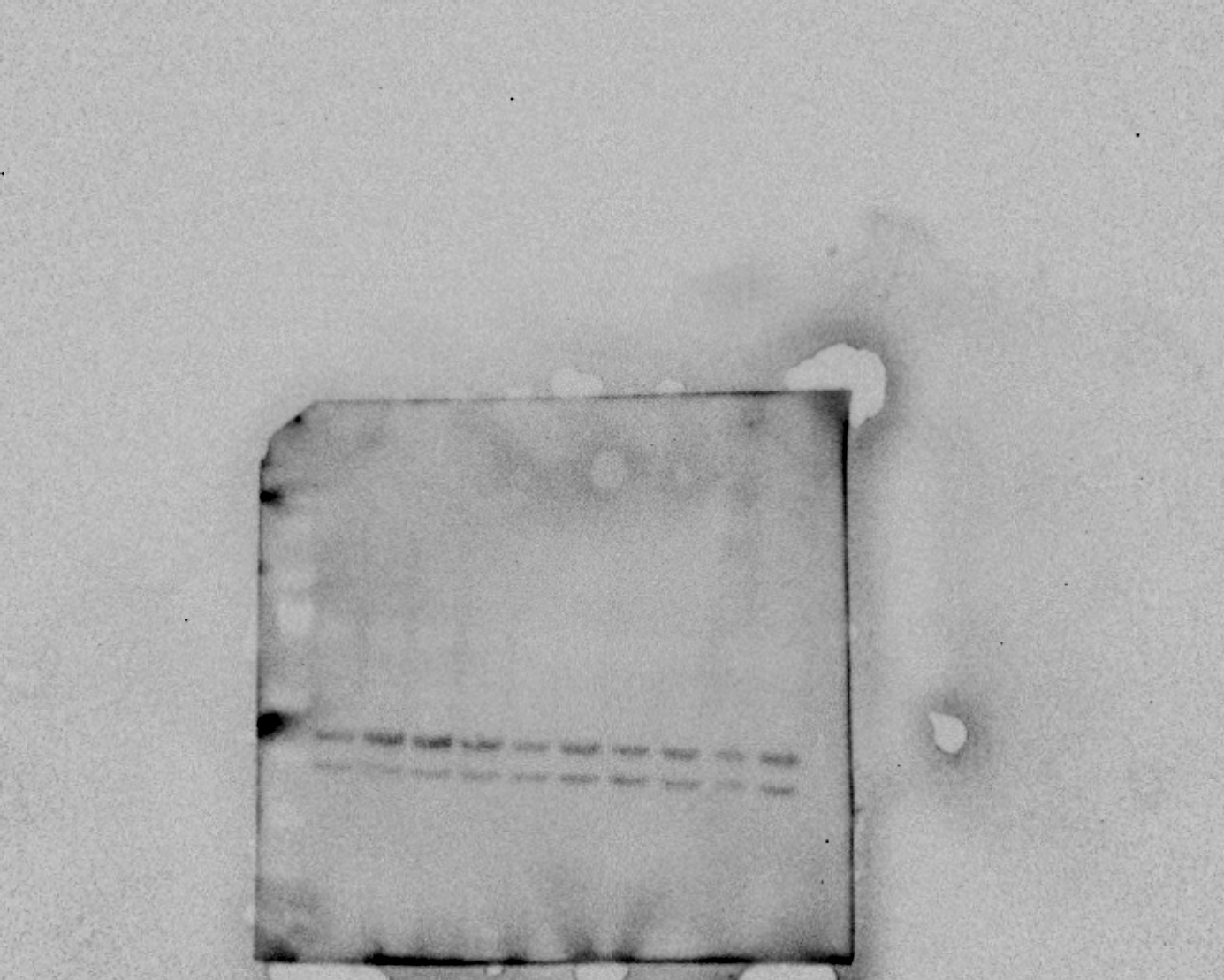

Supplement: Figure 5—source data 1. [file elife-73105-fig5-data1.zip › Figure 5-source data/Fig 5B/Fig5B_WB_GAPDH.tif]

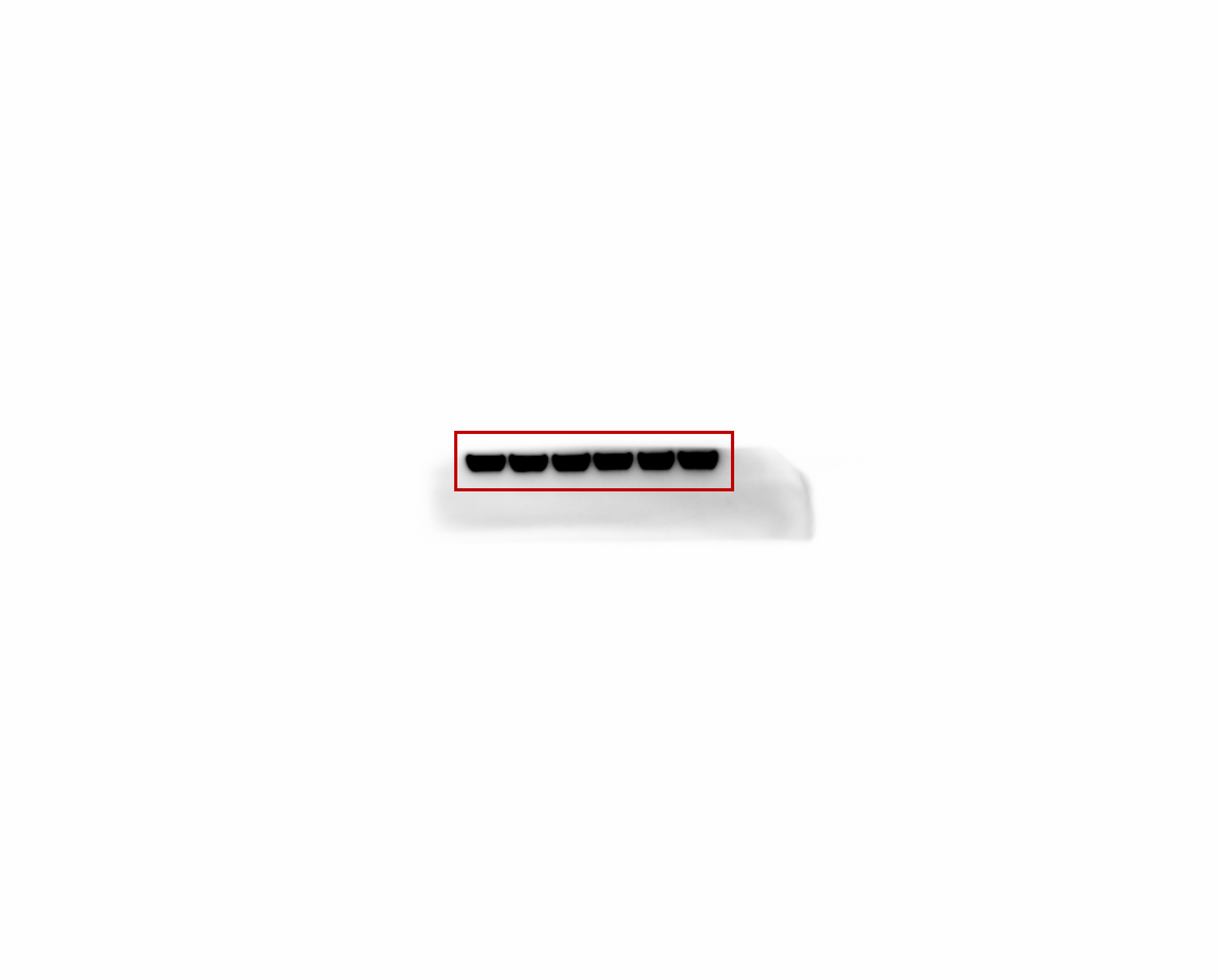

Supplement: Figure 7—source data 1. [file elife-73105-fig7-data1.zip › Figure 7-source data/Fig 7B/Fig 7B_WB_b-Actin-labeled.tif]

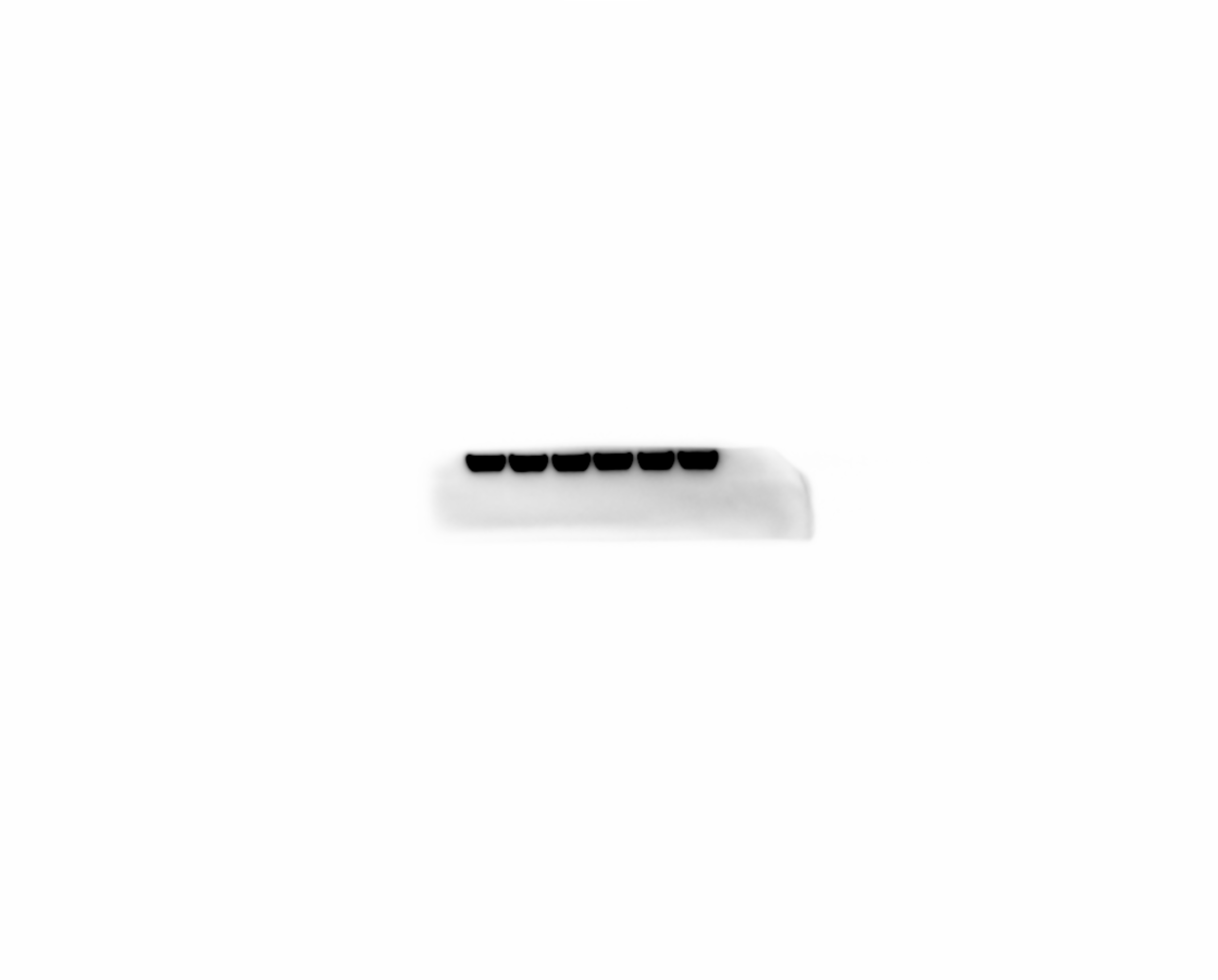

Supplement: Figure 7—source data 1. [file elife-73105-fig7-data1.zip › Figure 7-source data/Fig 7B/Fig 7B_WB_b-Actin.tif]

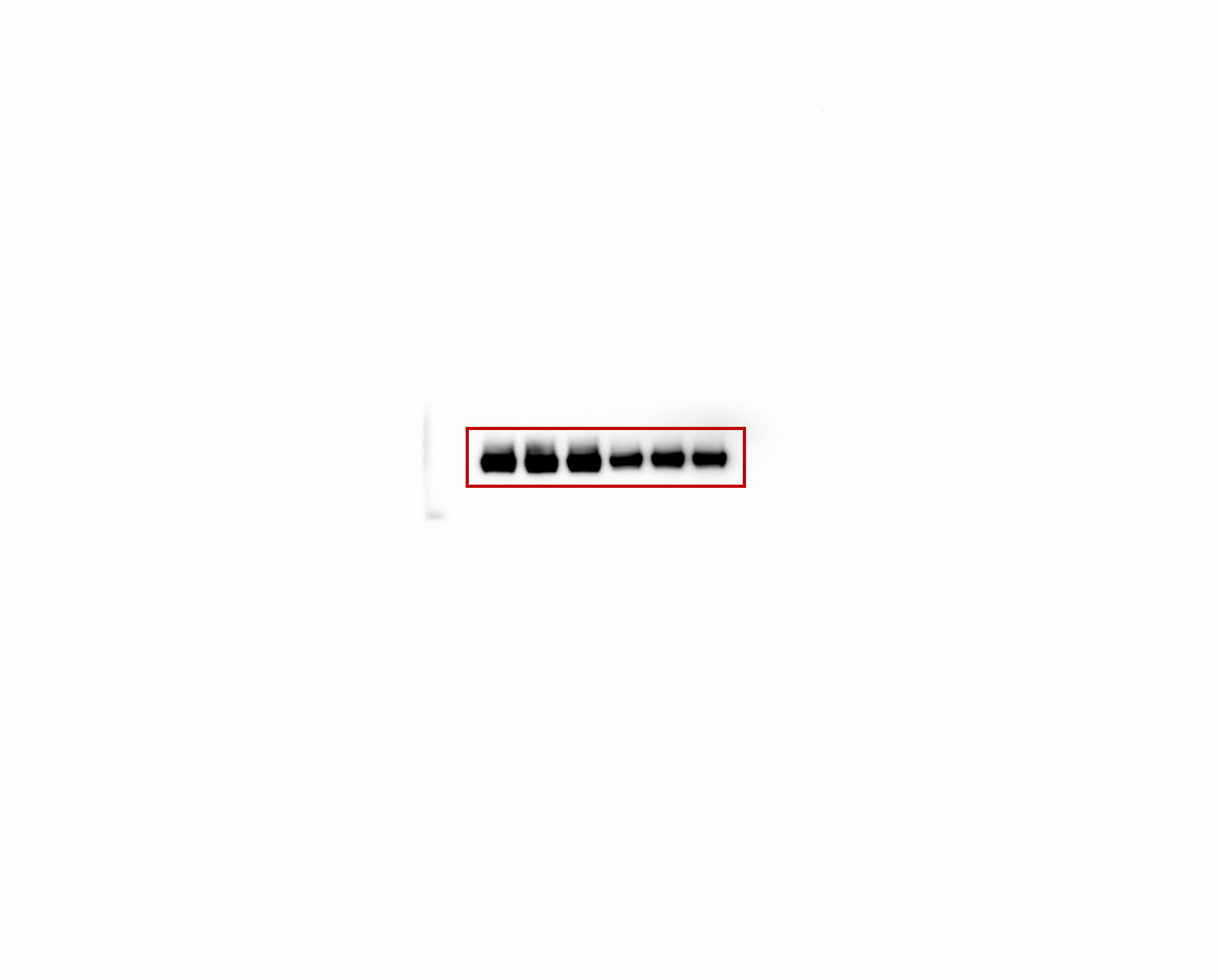

Supplement: Figure 7—source data 1. [file elife-73105-fig7-data1.zip › Figure 7-source data/Fig 7B/Fig 7B_WB_VEGFR2-labeled.tif]

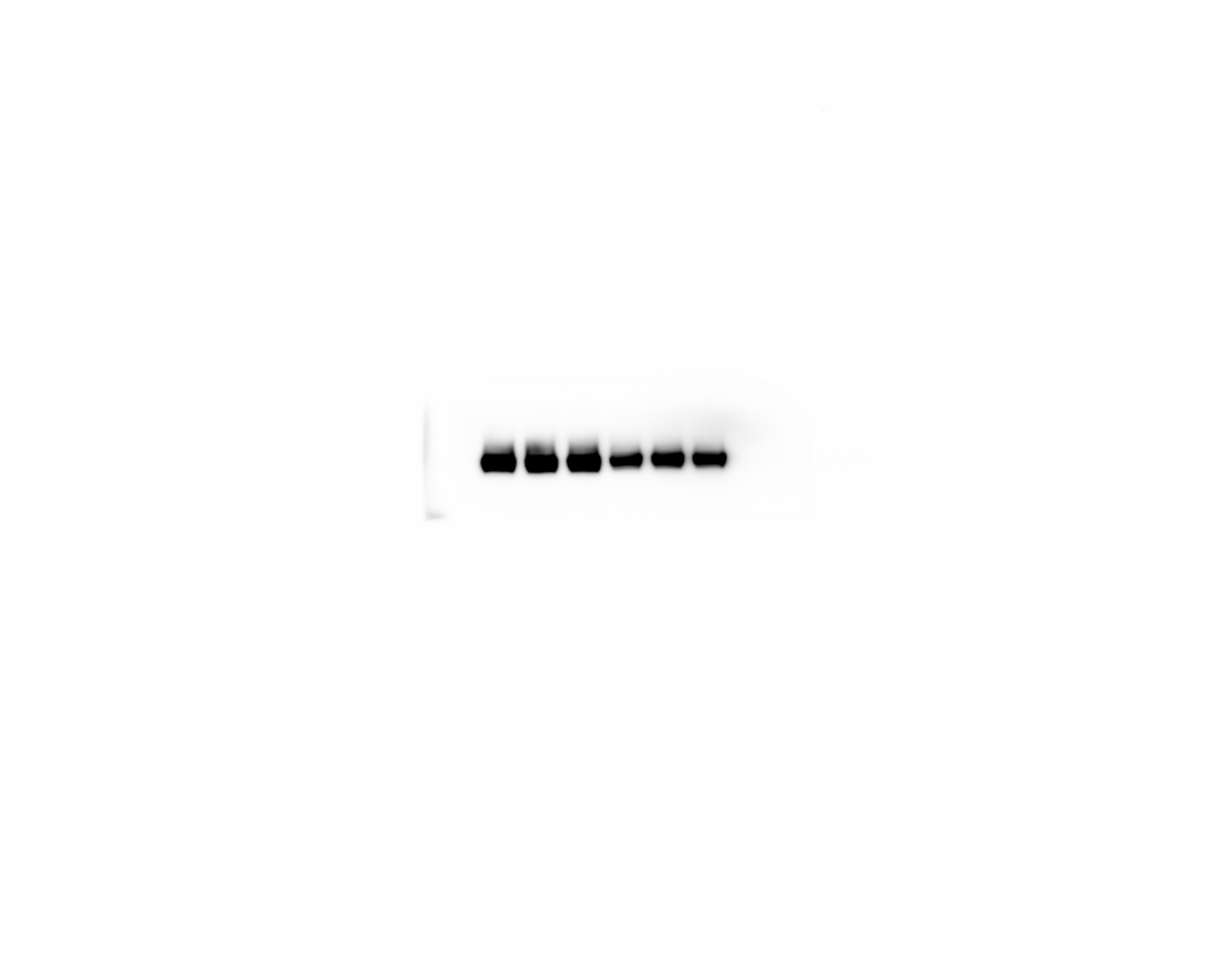

Supplement: Figure 7—source data 1. [file elife-73105-fig7-data1.zip › Figure 7-source data/Fig 7B/Fig 7B_WB_VEGFR2.tif]

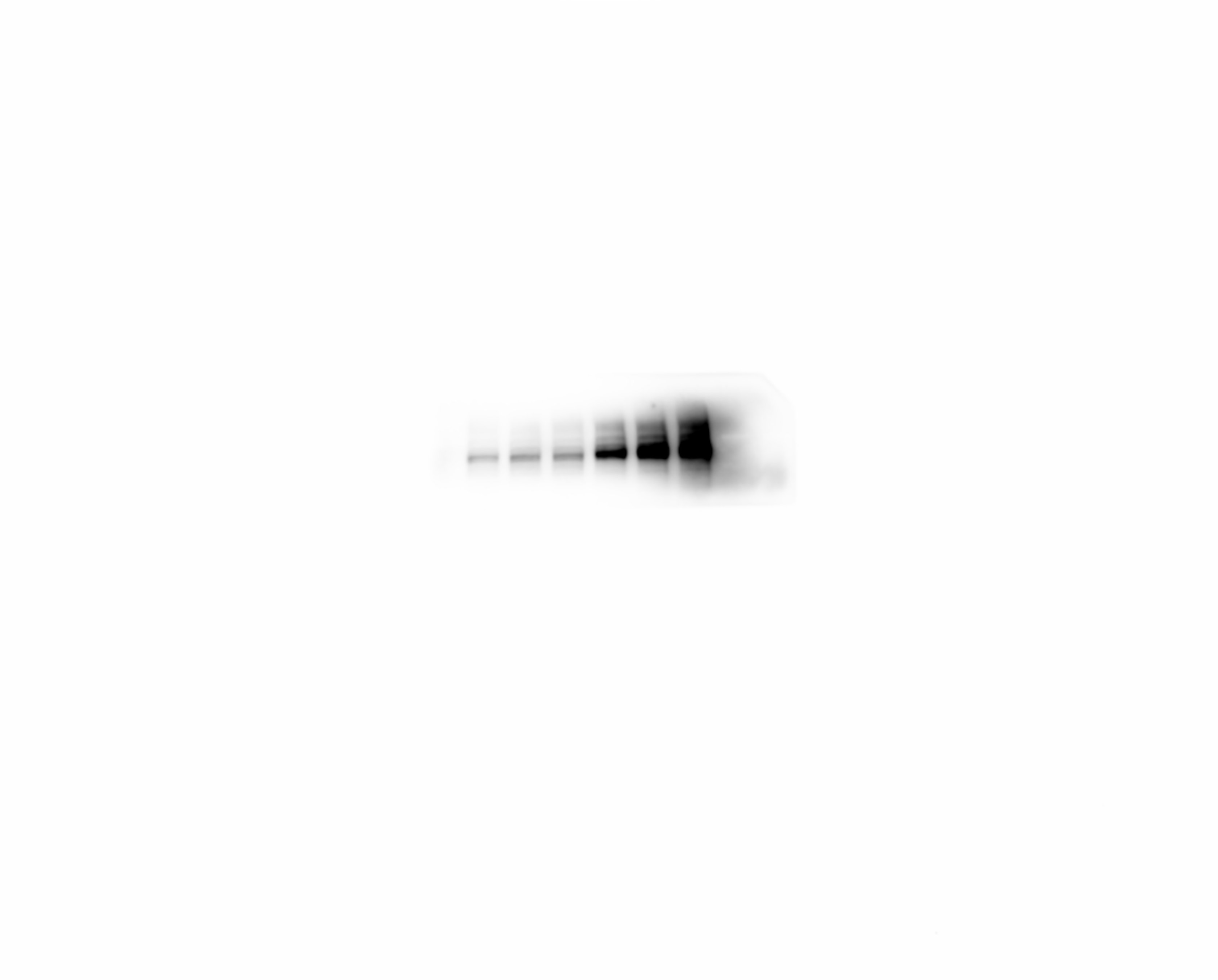

Supplement: Figure 7—source data 1. [file elife-73105-fig7-data1.zip › Figure 7-source data/Fig 7B/Fig 7B_WB_VEGFR1.tif]

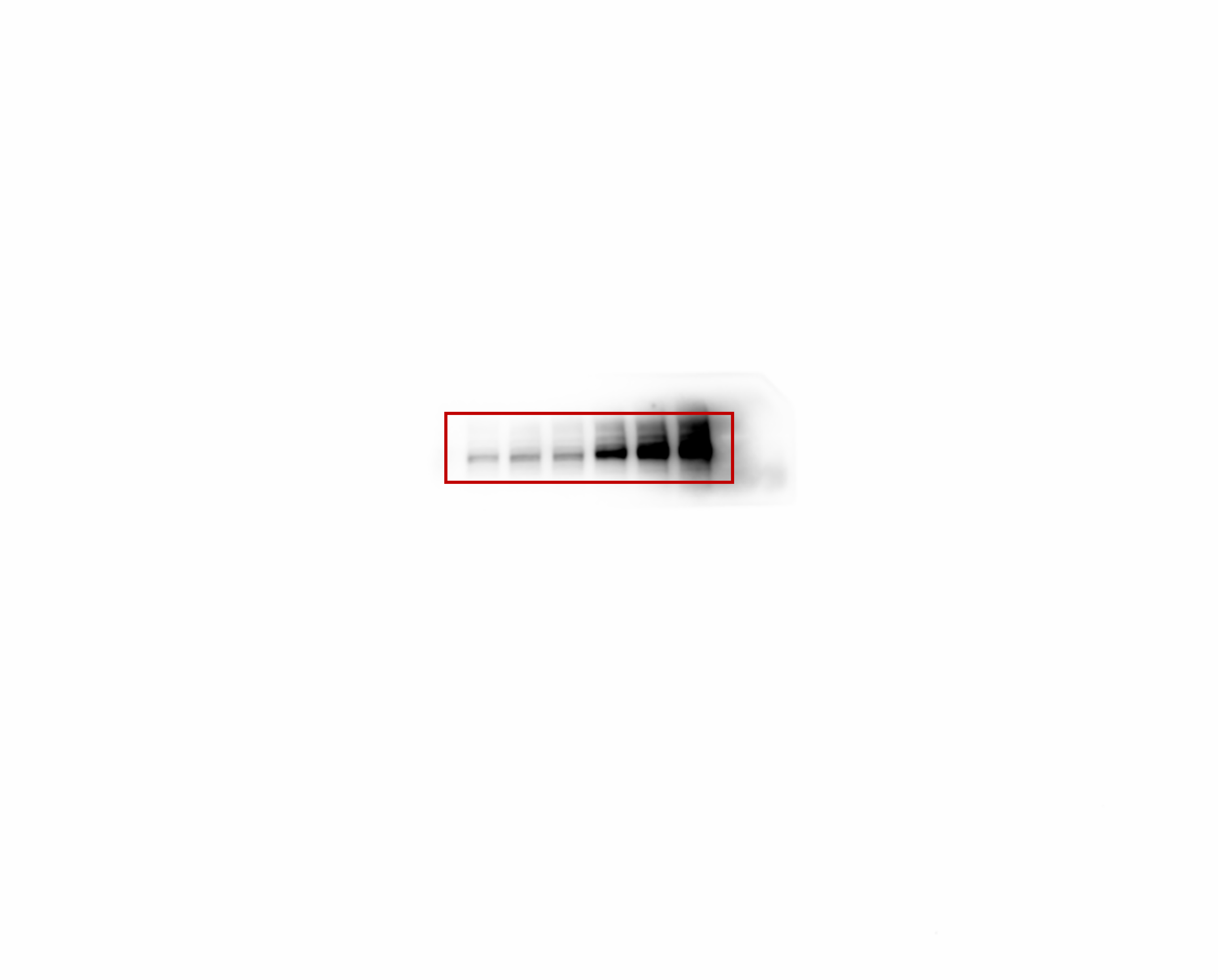

Supplement: Figure 7—source data 1. [file elife-73105-fig7-data1.zip › Figure 7-source data/Fig 7B/Fig 7B_WB_VEGFR1-labeled.tif]
